# Advances in Targeting BCR-ABL^T315I^ Mutation with Imatinib Derivatives and Hybrid Anti-Leukemic Molecules

**DOI:** 10.3390/molecules31020341

**Published:** 2026-01-19

**Authors:** Aleksandra Tuzikiewicz, Wiktoria Wawrzyniak, Andrzej Kutner, Teresa Żołek

**Affiliations:** 1 Department of Organic and Physical Chemistry, Faculty of Pharmacy, Medical University of Warsaw, 1 Banacha, 02-097 Warsaw, Poland; s089386@student.wum.edu.pl (A.T.); s086144@student.wum.edu.pl (W.W.); 2 Department of Drug Chemistry, Pharmaceutical and Biomedical Analysis, Faculty of Pharmacy, Medical University of Warsaw, 1 Banacha, 02-097 Warsaw, Poland; andrzej.kutner@wum.edu.pl

**Keywords:** BCR-ABL tyrosine kinase, T315I mutation, chronic myeloid leukemia, inhibitors, medicinal chemistry, targeted therapy, computational modeling, ligand-protein interactions

## Abstract

Resistance to imatinib remains a therapeutic challenge, largely driven by point mutations within the kinase domain of the *BCR-ABL*, among which the *T315I* substitution constitutes the most clinically significant barrier. Ponatinib effectively inhibits this mutant form but is limited by dose-dependent cardiovascular toxicity, prompting efforts to develop safer and more selective agents. Recent advances highlight aminopyrimidine-derived scaffolds and their evolution into thienopyrimidines, oxadiazoles, and pyrazines with improved activity against *BCR-ABL^T315I^*. Further progress has been achieved with benzothiazole–picolinamide hybrids incorporating a urea-based pharmacophore, which benefit from strategic hinge-region substitutions and phenyl linkers that enhance potency. Parallel research into dual-mechanism inhibitors, including Aurora and *p38* kinase modulators, demonstrates additional opportunities for overcoming resistance. Combination strategies, such as vorinostat with ponatinib, provide complementary therapeutic avenues. Natural-product-inspired approaches utilizing fungal metabolites provided structurally diverse scaffolds that could engage sterically constrained mutant kinases. Hybrid molecules derived from approved *TKIs*, including GNF-7, olverembatinib, and HG-7-85-01, exemplify rational design trends that balance efficacy with improved safety. Molecular modeling continues to deepen understanding of ligand engagement within the *T315I*-mutated active site, supporting the development of next-generation inhibitors. In this review, we summarized recent progress in the design, optimization, and biological evaluation of small molecules targeting the *BCR-ABL^T315I^* mutation.

## 1. Introduction

Chronic Myeloid Leukemia (CML) is a hematological malignancy driven by the uncontrolled expansion of aberrant myeloid cells. Its defining molecular hallmark is the Philadelphia chromosome (Ph), generated by a reciprocal translocation between the ABL proto-oncogene on chromosome 9 and the BCR gene on chromosome 22, designated as t(9;22)(q34;q11). This translocation produces the BCR-ABL fusion gene, which encodes a constitutively active tyrosine kinase that is both necessary and sufficient for CLM initiation and maintenance [1]. The progression of CML is further shaped by dysregulation of multiple signaling pathways, including the overexpression of histone deacetylases (HDACs), which suppress tumor suppressor and pro-apoptotic genes. Both BCR-ABL activity and HDAC-mediated epigenetic alterations play central roles in CML pathogenesis [2]. The BCR-ABL fusion gene is detected in nearly all cases of CML, 25–50% of adult B-lineage acute lymphoblastic leukemia (B-ALL), and approximately 5% of acute myeloid leukemia (AML), underscoring its broad relevance in hematological malignancies [3].

The development of small-molecule inhibitors targeting the BCR-ABL oncoprotein marked a major advance in the treatment of CML, transforming a previously fatal myeloproliferative disorder into a manageable chronic condition. Imatinib was the first ATP-competitive tyrosine kinase inhibitor (TKI), demonstrated outstanding clinical activity, and established the foundation for targeted therapy in CML [4]. Due to its favorable therapeutic index and durable molecular responses, imatinib continues to serve as a reference first-line agent. However, its long-term effectiveness is limited by the development of acquired resistance, which occurs in approximately 40% of patients. Early attempts to overcome imatinib resistance in CML focused on multi-drug strategies combining imatinib with interferon-alpha or cytarabine [5]. Subsequent studies explored combination therapies aimed at enhancing the antiproliferative activity of imatinib. In the FaDu squamous cell carcinoma (SCC) model, co-treatment with imatinib and vitamin D metabolite—tacalcitol or synthetic vitamin D analog PRI-1906—yielded synergistic or additive effects [6]. Similarly, in human leukemia HL-60 cells, tacalcitol augmented the antiproliferative activity of imatinib, with further potentiation observed upon the addition of docetaxel or cisplatin, primarily through cell-cycle arrest rather than apoptosis [7]. In A549 non-small cell lung cancer (NSCLC) cells, imatinib exhibited tumor growth inhibition only when combined with tacalcitol or synthetic 5,6-*trans*-calcipotriol (PRI-2205), progressing from additive to synergistic activity during prolonged exposure [8,9]. These findings suggested that imatinib and vitamin D compounds may modulate overlapping signaling pathways. Enhanced anticancer and anti-angiogenic activity was further demonstrated in triple-combination regimens incorporating calcitriol or tacalcitol with imatinib and cytotoxic agents, resulting in improved therapeutic responses and the potential for dose reduction in cisplatin or docetaxel [10]. The observed effects were associated with the downregulation of vascular endothelial growth factor A (VEGF-A), indicating that angiogenesis suppression may contribute to the mechanism of action.

**Figure 1:**
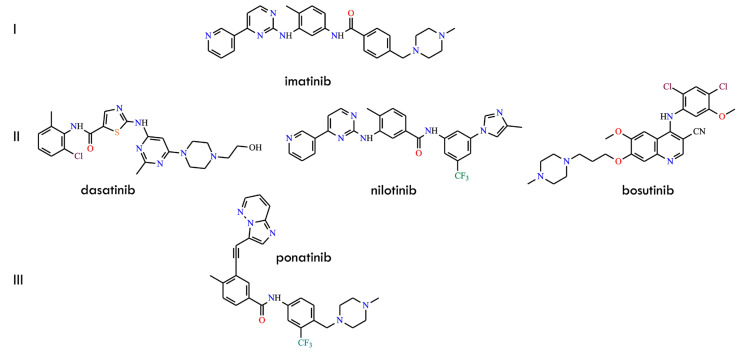
Structural formulas of initial generations of BCR-ABL kinase inhibitors ((**I**): imatinib; (**II**): dasatinib; nilotinib; and bosutinib; (**III**): ponatinib) (All structures were drawn using ChemWindow 6.0 software).

However, it has not been conclusively demonstrated that multi-drug regimens can effectively overcome imatinib resistance. The predominant mechanism of resistance involves point mutations within the BCR-ABL kinase domain that reduce inhibitor binding affinity or modify the conformational state of the active site. Although numerous resistance-associated substitutions have been identified, the gatekeeper mutation T315I—detected in 2–20% of resistance cases—remains one of the most challenging clinically [11,12,13]. This substitution disrupts a key hydrogen bond with imatinib, introduces steric hindrance that prevents binding of most first- and second-generation TKIs, and stabilizes the ABL DFG-in conformation. Consequently, imatinib, dasatinib, nilotinib, and bosutinib (Figure 1), as well as most other ATP-competitive inhibitors, exhibit markedly reduced potency against T315I-positive BCR-ABL variants [3,14,15]. Structure-guided drug design efforts ultimately yielded third-generation inhibitors such as ponatinib (Figure 1). Its ethynyl linker and optimized hinge-binding region enable accommodation of the bulkier isoleucine residue at position 315 while maintaining essential hydrophobic or van der Waals interactions. Further rational refinement targeting conformational rigidity, hydrophobic pocket occupancy, and solubility has improved selectivity and activity against both wild-type and mutant BCR-ABL. Additionally, hybrid inhibitors integrating structural elements from dasatinib, nilotinib, and ponatinib have been developed, producing molecules with enhanced activity against highly refractory single and compound mutants.

Beyond ATP-competitive inhibitors, hybrid molecules combining pharmacophores from dasatinib, nilotinib, and ponatinib have been explored to overcome mutation-driven resistance. Additionally, dual-function inhibitors targeting BCR–ABL and other critical proteins, such as Aurora kinases [16], JAK/STAT regulators [17], or HDACs [18], aim to simultaneously disrupt multiple signaling pathways essential for CML cell survival, potentially improving therapeutic efficacy. These multifunctional agents reflect an emerging design philosophy in which kinase inhibition is integrated with broader pathway modulation to achieve deeper molecular remissions. By 2018, the field reached a perceived plateau: resistance mechanisms were well characterized, encompassing single and compound kinase-domain mutations, activation of alternative signaling pathways, quiescent leukemic stem cell reservoirs, and BCR-ABL1-independent mechanisms [3]. Concurrently, treatment-free remission (TFR) became attainable in 40–60% of patients maintaining deep molecular responses (DMRs) under long-term therapy [19,20,21]. However, the past seven years have witnessed an unprecedented surge in both chemical innovation and clinical outcomes, driven by three transformative paradigms that extend far beyond classical ATP-competitive inhibition [22].

**Figure 2:**
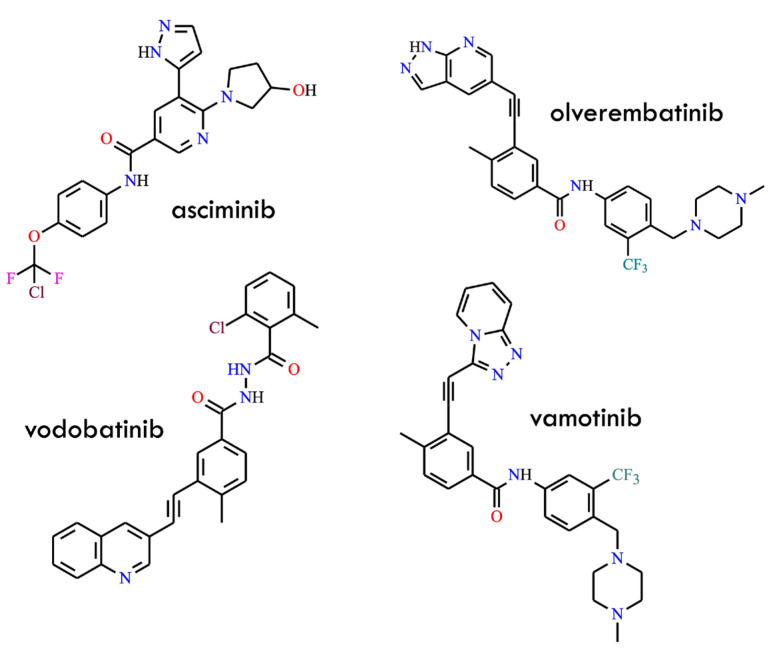
Structural formulas of the third generation of BCR-ABL kinase inhibitors: asciminib, olverembatinib, vodobatinib and vamotinib (All structures were drawn using ChemWindow 6.0 software).

First, the approval of asciminib (Figure 2) in 2021 introduced the first allosteric BCR-ABL1 inhibitor, which binds to the myristoyl pocket rather than the orthosteric ATP-binding site [23]. By stabilizing the auto-inhibited conformation of ABL1, asciminib effectively circumvents virtually all clinically relevant ATP-site mutations—including those involving T315I-inclusive compound mutations—while exhibiting a markedly cleaner “kinome profile” and minimal vascular toxicity. Its high selectivity, favorable pharmacokinetic properties, and reduced off-target vascular effects clearly distinguish it from earlier generations of TKIs. Moreover, the unique allosteric mechanism of asciminib enables combination strategies pairing allosteric and ATP-competitive inhibitors, which synergistically suppress the emergence of resistant clones [24,25]. Importantly, accumulating preclinical and clinical evidence further underscores its potential to deepen molecular responses and delay resistance evolution. Second, a new wave of fourth-generation ATP-competitive inhibitors has emerged, comprising molecules such as olverembatinib (HQP135, GZD-824) [26], vodobatinib (no efficacy against the T315I mutation) [27], vamotinib (PF-114) [28], and TERN-701 (HS-10382; chemical structure undisclosed) [29] (Figure 2).

These agents incorporate strategically engineered “cardiac safety-by-design” features: attenuated VEGF/PDGFR inhibitory activity, reduced lipophilicity, refined hinge-binding motifs, improved aqueous solubility, and minimal activation of pro-thrombotic signaling pathways [26]. Such modifications aim to preserve ponatinib-like potency against T315I and compound mutants while mitigating arterial occlusive events. Many of these inhibitors demonstrate robust target engagement, enhanced selectivity profiles, and tolerability superior to earlier high-potency TKIs. In addition, their optimized physicochemical properties contribute to more favorable pharmacokinetic behavior and reduced off-target toxicity, aligning with contemporary safety expectations in kinase inhibitor design.

Third, entirely new chemical modalities have entered both preclinical pipelines and early clinical development. BCR-ABL1-targeted PROTACs (proteolysis-targeting chimeras) and molecular glue degraders represent a mechanistically distinct strategy—redirecting the oncogenic kinase for ubiquitination and proteasomal degradation rather than competitive inhibition [30]. Covalent-allosteric hybrid inhibitors similarly introduce an expanded mechanistic paradigm by irreversible target engagement with conformational modulation of the myristoyl pocket, thereby enabling potent suppression of multidrug-resistant variants. Additional innovative strategies include transcriptional and translational inhibitors that downregulate BCR-ABL1 mRNA or protein synthesis, as well as immunotherapeutic approaches such as BCR-ABL1-derived peptide vaccines and CAR-T constructs. These next-generation modalities aspire not only to inhibit but to eliminate the leukemogenic driver, potentially targeting leukemic stem cell reservoirs responsible for molecular persistence and relapse [31,32]. Collectively, these approaches broaden the therapeutic landscape and provide avenues for addressing resistance mechanisms that remain incompletely controlled by ATP-competitive or allosteric TKIs. Asciminib is increasingly considered a contender for first-line therapy. Dual-inhibition combinations are achieving unprecedented rates of molecular response (MR^4.5^, defined as a ≥4.5 log reduction in BCR-ABL1 transcript levels) and sustained treatment-free remission. The concept of a pharmacological cure—once regarded as aspirational—is now supported by emerging clinical evidence [33]. These developments underscore a paradigm shift toward personalized, mechanism-informed treatment strategies in chronic myeloid leukemia.

This review provides an in-depth analysis of recent progress in BCR-ABL inhibitor development, with particular emphasis on third-generation compounds engineered to overcome T315I and other clinically significant resistance mutations. Detailed structure-activity relationship (SAR) trends, pharmacophore optimization strategies, and innovative approaches for resolving drug resistance in CML are discussed within the broader context of medicinal chemistry and targeted therapy. Taken together, the presented evidence highlights both the maturation of established therapeutic concepts and the growing impact of novel chemical modalities on the future trajectory of BCR-ABL1-directed drug discovery.

## 2. DFG-in and DFG-Out Conformations of BCR-ABL Kinase

**Figure 3:**
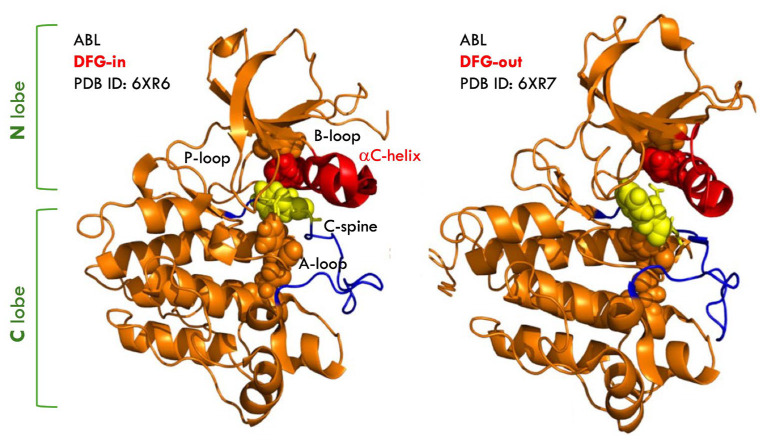
Schematic representation of the stable, fully active ground state of the ABL kinase domain (PDB ID:6XR6) (**left**), and the inactive state I1 (PDB ID: 6XR7) (**right**). The ABL structures are shown as orange ribbons. The αC-helix (residues 291–311) is shown as red ribbons, the A-loop (residues 398–421) in blue, and the DFG-motif (residues 400–402) as yellow sticks. The C-spine residues M309, L320, H380, F401, and D440 are shown as yellow spheres (Figure was generated using Discovery Studio 2025 (BIOVIA, San Diego, CA, USA)).

The kinase domain comprises an N-terminal lobe (N-lobe) and a C-terminal lobe (C-lobe), connected by a flexible hinge region, with three conserved structural elements: the activation loop (A-loop), the Asp-Phe-Gly (DFG) motif, and the αC helix (Figure 3). The DFG motif plays a central role in governing kinase activation and catalytic competence. BCR-ABL inhibitors are commonly classified as DFG-in or DFG-out based on how they engage the kinase domain [34]. The DFG-in state represents the active conformation of the kinase, in which the Asp (D) residue is oriented toward the catalytic site to coordinate Mg^2+^ and support proper ATP positioning. In contrast, the DFG-out state corresponds to an inactive conformation utilized by several type II inhibitors, characterized by rotation of the Phe (F) residue into the ATP-binding pocket and displacement of Asp outward. These conserved features complicate the design of selective inhibitors due to structural homology across the kinase family. Imatinib is a DFG-out inhibitor that stabilizes the kinase in an inactive conformation.

In contrast, dasatinib, a DFG-in inhibitor, binds the kinase in a conformation resembling the active ATP-bound state. Nilotinib, another DFG-out inhibitor, was developed to improve potency against imatinib-resistant mutants. Both inhibitors form a key hydrogen bond with the gatekeeper residue Thr315, which is critical for affinity and selectivity. The T315I mutation disrupts this interaction, introducing steric hindrance, rendering these inhibitors ineffective against the mutant. To overcome T315I-mediated resistance, inhibitors must avoid reliance on the Thr315 hydrogen bond and exploit alternative interactions within the kinase. Recent structural and biophysical evidence suggests that further indices that inhibitor selectivity is shaped not only by DFG-out stabilization but also by slow conformational transitions occurring after ligand binding. Only kinases capable of adopting the DFG-out state can accommodate imatinib, highlighting the importance of activation-loop flexibility. Experimental and computational studies demonstrate that the DFG-in state is more populated at equilibrium and that interconversion between DFG-in and DFG-out occurs on the millisecond timescale. These dynamics are essential for enzymatic function and inhibitor binding, emphasizing the need to consider both structural and kinetic factors in drug design [35].

## 3. Imatinib Resistance and New Active Derivatives

In the treatment of CML, resistance to imatinib emerges frequently, particularly in advanced disease stages and among older patients. This resistance arises from multiple mechanisms, including point mutations within the BCR-ABL kinase domain, amplification of the BCR-ABL gene, activation of BCR-ABL-independent pathways, variable plasma bioavailability, and insufficient intracellular drug concentrations. More than 100 amino acid substitutions within the kinase domain have been identified as contributors to imatinib resistance; however, approximately 85% of clinically relevant cases involve seven hotspot positions (M244V, G250E, Y253F/H, E255K/V, T315I, M351T, and F359V). Consequently, intensive medicinal chemistry efforts have been directed toward the development of next-generation BCR-ABL inhibitors capable of overcoming these clinically significant mutations [36]. In the following section, recently reported imatinib derivatives are summarized and evaluated, with particular emphasis on their structural features, mechanistic rationale, and therapeutic potential.

### 3.1. Aminopyrimidine- and Thienopyrimidine-Based BCR-ABL Inhibitors Targeting T315I

**Figure 4:**
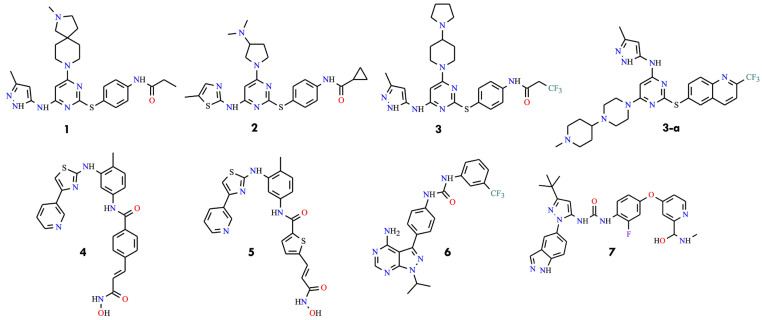
Structural formulas of early BCR-ABL inhibitors **1**–**7** and **3-a**, capable of overcoming the T315I resistance mutation (All structures were drawn using ChemWindow 6.0 software).

The development of BCR-ABL inhibitors capable of overcoming the T315I resistance mutation has been a major focus of contemporary chronic myeloid leukemia research. The first significant contribution came in 2008, when aminopyrimidine-based Aurora kinase inhibitors were reported exhibiting activity against both wild-type and T315I-mutated BCR-ABL. The initial series featured C2 thiophenyl-substituted aminopyrimidines with para-substituted phenyl rings and amino functionalities, often replaced at C4 or C6 with a 5-(3-methyl-1H-pyrazol) moiety. Representative compounds (**1**–**3**) (Figure 4) demonstrated inhibitory activity against both native and mutant forms [37].

A subsequent, similar series of aminopyrimidines, including compound **3-a** (Figure 4), achieved IC_50_ values below 25 nM, underscoring the potential of this scaffold to overcome T315I-mediated resistance [38]. Additionally, bifunctional inhibitors (**4** and **5**) were introduced that exhibited activity against T315I BCR-ABL (IC_50_ = 1–34 µM), representing an early effort to enhance target engagement through dual functional groups (Figure 4) [39]. Similarly, pyrazolo [3,4-d]pyrimidines were reported bearing C4 amino substitutions, achieving nanomolar IC_50_ values (e.g., compound **6**) (Figure 4) [40]. Moreover, rebastinib-related inhibitors were described with compound **7** demonstrating the highest potency against T315I, achieving an IC_50_ of 12 nM (Figure 4) [41].

**Figure 5:**
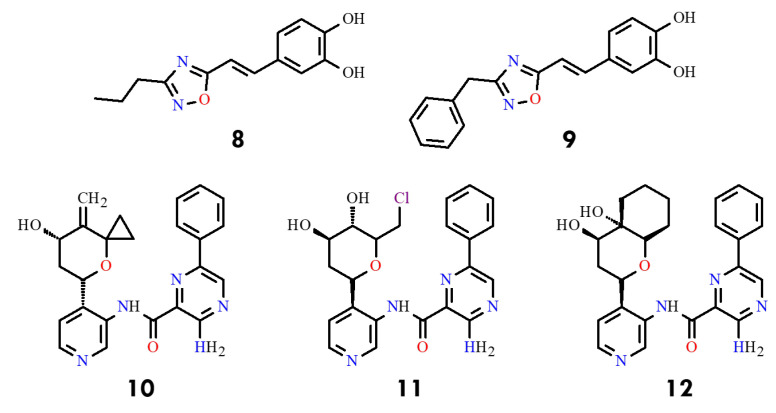
Structural formulas of compounds **8**–**12**, highlighting advances in the design of BCR-ABL inhibitors active against the T315I mutation (All structures were drawn using ChemWindow 6.0 software).

**Figure 6:**
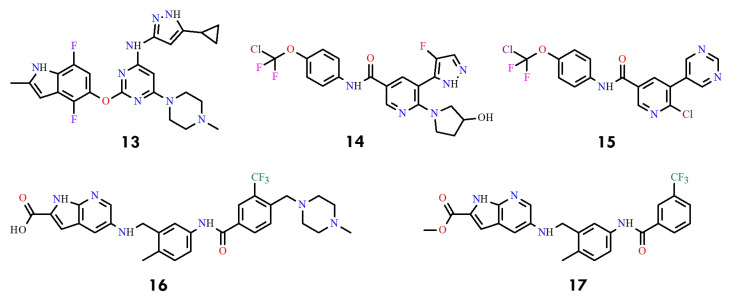
Structural formulas of compounds **13**–**17** targeting BCR-ABL^T315I^ (All structures were drawn using ChemWindow 6.0 software).

A series of 4-amino-substituted thieno [3,2-d]pyrimidines bearing a 7-carboxamide group was reported as potent BCR-ABL kinase inhibitors, with selected compounds exhibiting low-nanomolar activity (1–10 nM) against both wild-type and T315I-mutant BCR-ABL [42]. Another class of compounds, 1,2,4-oxadiazole derivatives, displayed moderate inhibitory activity, with IC_50_ values of 1.88–2.32 µM for compounds **8** and **9** (Figure 5). Subsequent work disclosed 3-amino-pyrazine-2-carboxylic acid pyridin-3-ylamide derivatives **10**–**12** incorporating oxygenated cyclic substituents at C4, achieving IC_50_ values of 0.03–0.07 µM against T315I (Figure 5). In contrast, indol-5-ol derivatives, such as compound **13** (NTW-3475) (Figure 6), further improved potency (IC_50_ = 0.1 µM) [43].

More recently, additional reports described two benzamide-based series with IC_50_ values ranging from 1.2 to 23 nM. Representative examples include compound **14** (IC_50_ = 0.8 nM) and compound **15** (IC_50_ = 2.0 nM), highlighting the systemic optimization of substituents to enhance potency, kinase selectivity, and pharmacokinetic properties [44,45] (Figure 6). Further studies disclosed acidic and ester analogs (compounds **16** and **17**) with IC_50_ values of approximately 10 µM, thereby expanding the chemical space for future inhibitor refinement [46,47].

Collectively, these efforts illustrate the progressive evolution of aminopyrimidine- and thienopyrimidine-derived scaffolds and highlight ongoing structural innovations aimed at addressing the clinically challenging T315I mutation while maintaining selectivity and favorable pharmacological profiles.

### 3.2. Development and Optimization of HS-438-Derived Benzothiazole–Picolinamide Hybrids Targeting BCR-ABL^T315I^

**Figure 7:**
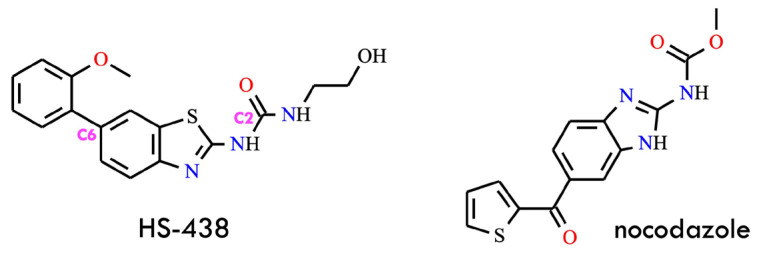
Structural formulas of HS-438 and nocodazole (All structures were drawn using ChemWindow 6.0 software).

A BCR-ABL inhibitor, HS-438 [1-(2′-hydroxyethyl)-3-(6-(2-methoxyphenyl)benzo[d]thiazol-2-yl)urea] (Figure 7), was synthesized and evaluated for its anti-leukemic potential both in vitro and in vivo. The compound was rationally designed as a structurally modified derivative of nocodazole, a well-known microtubule inhibitor. Previous studies demonstrated that the nocodazole scaffold can induce apoptosis in CML cells and exhibit inhibitory activity against the T315I BCR-ABL1 mutation. Based on these observations, nocodazole was selected as a lead structure, and a series of systematic structural optimizations were introduced to enhance its overall pharmacological profile.

The optimization process began at the C2 position, where the terminal methoxy group was replaced with an ethylamine moiety, resulting in the formation of an ethylurea substructure. Subsequent analyses confirmed that this ethylamine-derived functional group was essential for maintaining inhibitory potency. Additional modifications at the C6 position introduced a benzene ring, which was predicted to engage in favorable van der Waals interactions with the Ile315 residue, thereby mitigating the steric hindrance imposed by the T315I gatekeeper mutation. Further scaffold refinement involved substituting the original imidazole ring with a thiazole ring. Although benzothiazole derivatives have been previously reported to enhance biological activity, in this specific context, the resulting level of enzymatic inhibition was insufficient. Consequently, additional optimization steps were undertaken, including the incorporation of water-soluble substituents on the ethylurea moiety. These modifications improved the compound’s inhibitory properties, ultimately yielding HS-438, which demonstrated potent activity against the T315I-mutated kinase. Notably, HS-438 showed lower toxicity than ponatinib, underscoring its potential as a promising therapeutic candidate for overcoming T315I-driven resistance in CML [22]. HS-438 exhibited potent inhibitory activity against BCR-ABL^T315I^, a mutant form resistant to imatinib. Mechanistic studies demonstrated that treatment with HS-438 induced cell-cycle arrest at the G_0_/G_1_ phase and effectively triggered apoptotic cell death in BCR-ABL-expressing leukemia cells. In a Ba/F3 T315I xenograft mouse model, HS-438 significantly suppressed tumor growth, whereas imatinib was ineffective against the resistant mutation [48]. The *N*-methylpicolinamide fragment has been identified as a privileged motif in various anticancer kinase inhibitors, including sorafenib, regorafenib, and BLZ-945, where it contributes to hinge binding and favorable drug-like properties. Notably, prior to this work, no derivatives containing the *N*-methylpicolinamide motif had exhibited activity against BCR-ABL^T315I^, underscoring the value of this scaffold as a foundation for further inhibitor design.

**Figure 8:**
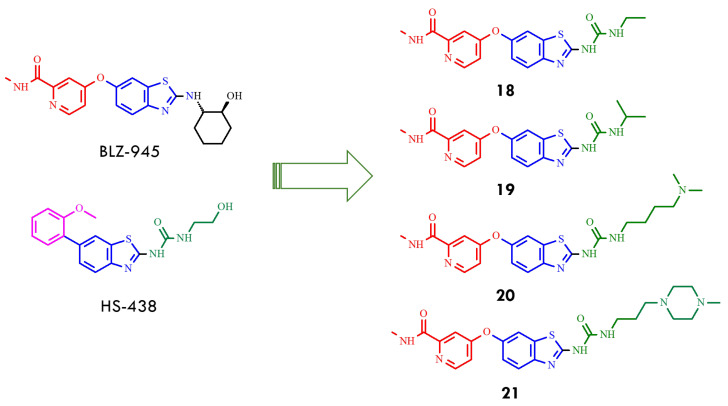
Structural formulas of benzothiazole–picolinamide hybrids (**18**–**21**) resulted from hybridization of benzothiazole–picolinamide BLZ-945 and urea derivative HS-438 (All structures were drawn using ChemWindow 6.0 software, based on data from ref. [49]).

Consequently, a new series of benzothiazole–picolinamide hybrids was developed, retaining the 2-benzothiazolyl urea core of HS-438 while incorporating a picolinamide ether at the C6 position to enhance hydrogen bonding in the hinge region and improve binding affinity. In parallel, systematic modifications of the C2 aliphatic urea side chain were undertaken by varying chain length and terminal substituents. Among the first two analogs, the ethylurea derivative (**18**) exhibited greater potency than the isopropyl analog (**19**) (Figure 8), with IC_50_ values of 33 nM versus 115 nM against BCR-ABL^T315I^. Substitution of the urea moiety with a thiourea group resulted in a 3–6-fold loss of potency, highlighting the essential role of the urea functionality in maintaining inhibitor activity [49].

**Figure 9:**
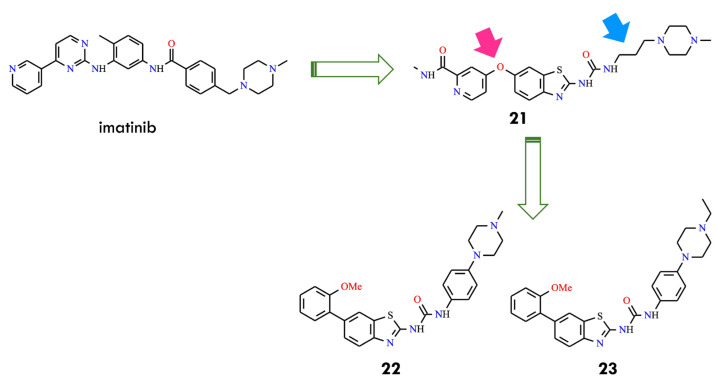
Rational design of BCR-ABL inhibitors **22** and **23** through replacement of the oxypicolinamide moiety with a 2-methoxyphenyl group (pink arrow) and substitution of the propyl linker with a phenyl group in compound **21** (blue arrow) (All structures were drawn using ChemWindow 6.0 software, based on data from ref. [50]).

Further optimization of the benzothiazole–picolinamide series focused on extending the urea side chain and introducing cyclic amine substituents to improve both potency and pharmacokinetic properties. Among the urea derivatives, the *N,N*-dimethylamine butylurea analog **20** demonstrated high potency, achieving IC_50_ values of 24.9 nM and 55.9 nM under different assay conditions. In contrast, within the propylurea series, the *N*-methylpiperazine derivatives **21** exhibited the most favorable balance of activity and solubility, with an IC_50_ of 39.9 nM against BCR-ABL^T315I^ (Figure 8). These findings indicated that the presence of *N*-methylpiperazine and *N,N*-dimethylamine substituents not only enhanced aqueous solubility but also reinforced binding interactions within the kinase active site. SAR analysis further revealed that propyl and butyl linkers generally provide superior activity compared with shorter ethyl linkers, underscoring the relevance of side-chain length and conformational flexibility for optimizing target engagement. Collectively, compounds **20** and **21** (AKE-5l) were identified as the most potent derivatives within this series, with **21** emerging as the lead compound [49]. Subsequent efforts focused on additional modifications to the benzothiazole–picolinamide scaffold to obtain analogs with improved efficacy relative to **21**, thereby extending the therapeutic potential of this chemotype against T315I-mediated resistance. These studies underscore the value of rational design and SAR-guided optimization in the development of next-generation BCR-ABL inhibitors capable of addressing clinically relevant resistance mutations. The integration of hinge-region interactions, strategic side-chain modification, and tailored substituent selection provides a strong foundation for advancing potent inhibitors targeting resistant leukemia variants. Subsequent optimization focused on replacing the 6-oxypicolinamide moiety of **21** with a 2-methoxyphenyl group, inspired by the higher activity observed for HS-438 (IC_50_ = 0.064 nM), and substituting the flexible propyl linker with a rigid phenyl ring to enhance structural rigidity and enable potential π–π interactions. Among the resulting analogs, compounds **22** and **23** (AK-HW-90), bearing terminal nitrogen substituents, demonstrated the most pronounced improvement in activity (Figure 9). Notably compound **23**, featuring an N-ethylpiperazine group, exhibited sub-nanomolar potency (IC_50_ = 0.651 nM) against BCR-ABL^T315I^, surpassing both **21** (IC_50_ = 39.9 nM) and **22** (IC_50_ = 2.0 nM) [50]. These data indicate that even subtle structural refinements—especially those located at the hinge region and within the linker architecture—can substantially enhance binding affinity and selectivity toward T315I-mutant kinase. Guided by the exceptional inhibitory profile of HS-438, the 2-methoxyphenyl substituent was subsequently incorporated at the C6 position of the benzothiazole core to further improve potency relative to the 6-oxypicolinamide group.

At the same time, the flexible propyl linker connecting the ureidobenzothiazole and *N*-methylpiperazine fragments in **21** was replaced with a rigid phenyl spacer, intended to overall molecular planarity, extend π-conjugation, and strengthen interactions within the ATP-binding pocket. Further modifications included functionalization of the terminal phenyl ring with various hydrophilic and lipophilic substituents to explore their impact on kinase inhibition and cellular selectivity. Comprehensive biological evaluation confirmed that the combined presence of the ortho-methoxyphenyl group, a phenyl linker, and terminal nitrogen substituents produced a synergistic enhancement of BCR-ABL inhibitory potency. Comparison of **21** and **23** highlights the critical contribution of electronic and conformational tuning: although both maintain the core ureidobenzothiazole scaffold and an *N*-alkylpiperazine terminus, replacement of the oxypicolinamide moiety and propyl linker with a 2-methoxyphenyl substituent and phenyl bridge shifted the activity profile from a narrow, mutation-restricted effect to a broader spectrum across multiple cell types.

### 3.3. Dual Aurora/ABL Kinase Inhibition: Tozasertib as a Potent T315I-Active Agent

**Figure 10:**
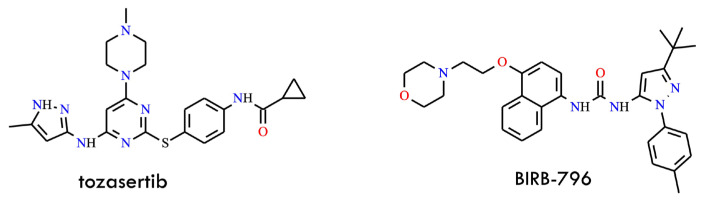
Structural formulas of tozasertib and BIRB-796 (The structures were drawn using ChemWindow 6.0 software).

Tozasertib (VX-680, MK-0457) (Figure 10) is a broad-spectrum Aurora kinase inhibitor that induces apoptosis in diverse tumor cell types by disrupting mitotic progression [51]. The Aurora kinase family, comprising Aurora A, B, and C, consists of serine/threonine kinases essential for accurate chromosomal segregation and cytokinesis during cell division. Dysregulated Aurora kinase activity is frequently observed in multiple human malignancies, underscoring their pivotal role in tumorigenesis [52]. Notably, tozasertib was subsequently identified as a dual-acting inhibitor, capable of targeting both Aurora kinases and ABL kinase-including the clinically challenging T315I gatekeeper mutant, which mediates resistance to most first- and second-generation TKIs.

Structural and biochemical studies demonstrated that tozasertib binds the ABL active site in a conformation accommodating the threonine-to-isoleucine substitution at residue 315, thereby maintaining high affinity for the mutant enzyme [53].

In biochemical assays, tozasertib inhibited ABL^T315I^ with an IC_50_ of approximately 30 nM, confirming its robust potency against this otherwise refractory mutant. In contrast, BIRB-796 (doramapimod) (Figure 10), a chemically unrelated kinase inhibitor, exhibited an inverse selectivity profile, showing stronger inhibition of BCR-ABL^T315I^-expressing cells (IC_50_ = 2–3 μM) than of wild-type cells (IC_50_ ≈ 10 μM). This complementary inhibitory pattern distinguishes tozasertib and BIRB-796 from imatinib, dasatinib (BMS-354825), and other classical ABL inhibitors, which display minimal activity against T315I [54]. Cell-based studies further confirmed that tozasertib suppresses the proliferation of both wild-type and mutant BCR-ABL-expressing cells while significantly reducing BCR-ABL autophosphorylation, demonstrating effective target engagement in a cellular context. Consistent with its Aurora kinase activity, tozasertib also disrupts mitotic progression, highlighting its dual mechanism of action: cell-cycle perturbation via Aurora kinase blockade combined with direct inhibition of ABL^T315I^ signaling. Collectively, these findings position tozasertib as an early and mechanistically distinctive example of a dual Aurora/ABL kinase inhibitor capable of overcoming T315I-mediated resistance, exhibiting biochemical potency comparable to that of later-generation ABL inhibitors and a binding profile complementary to classical TKIs.

## 4. Hybrid BCR-ABL Inhibitors: Design Strategies and Preclinical Evaluation

Numerous studies have focused on the development of hybrid molecules derived from existing BCR-ABL inhibitors, aiming to overcome resistance associated with point mutations. These hybrid structures often incorporate key pharmacophoric elements from dasatinib, nilotinib, or ponatinib, combining features of multiple inhibitors into a single molecule. Since the introduction of second-generation TKIs, several of these hybrid compounds have demonstrated promising inhibitory activity against both wild-type and mutant forms of BCR-ABL in preclinical studies. Despite their encouraging biochemical and cellular efficacy, none of these hybrids has yet progressed to widespread clinical application for the treatment of CML or ALL. Ongoing research continues to explore structure–activity relationships, aiming to enhance potency, selectivity, and pharmacokinetic properties to enable future clinical translation.

### 4.1. Ponatinib-Inspired BCR-ABL Inhibitors

**Figure 11:**
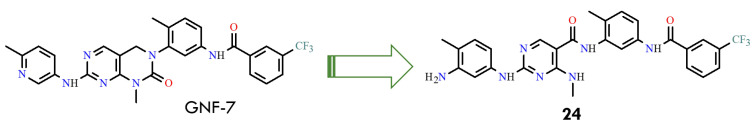
Discovery of the BCR-ABL inhibitor **24** (CHML-ABL053) by modifications of GNF-7 (All structures were drawn using ChemWindow 6.0 software).

A notable example of a ponatinib-inspired compound is GNF-7, which is based on a dihydropyrimidopyrimidine core scaffold and served as a lead for the discovery of the potent and selective BCR-ABL inhibitor **24** (CHMFL-ABL053) (Figure 11) [55]. GNF-7 functions as a type II BCR-ABL inhibitor, meaning it binds to the inactive DFG-out conformation of the kinase, stabilizing the enzyme in a catalytically inactive state. This binding mode enables the inhibitor to overcome the T315I gatekeeper mutation, which confers resistance to most first- and second-generation TKIs.

Despite its potency against BCR-ABL^T315I^, GNF-7 exhibits considerable off-target activity, including inhibition of JAK1-3, FGFR3, FLT3, PDGFR, and TRKC. Structural insights from molecular modeling revealed that the picoline moiety occupies the hinge-binding region, forming key hydrogen bonds, while the dihydropyrimidopyrimidine scaffold is positioned adjacent to the gatekeeper residue Thr315. The trifluoromethylbenzene group interacts with the hydrophobic pocket created by the DFG-out shift, contributing to binding affinity and selectivity. These structural observations highlight specific regions amenable to medicinal chemistry optimization, including the hinge-binding group, the gatekeeper-adjacent core, and the hydrophobic DFG pocket. Rational modifications in these regions can enhance potency against resistant mutants while reducing off-target effects and giving a framework for the next-generation BCR-ABL inhibitors with improved selectivity and efficacy against T315I-mediated drug resistance [55]. Among the 40 compounds investigated, compound **24** was highlighted in the study for exhibiting the most favorable activity and selectivity profile, with an IC_50_ of 70 nM against ABL1 kinase and 60 nM against p38α. However, disappointingly, **24** completely lost activity against the gatekeeper T315I mutant [55]. Other compounds, structurally more similar to GNF-7, were not evaluated on a broader scale, representing a potential avenue for future research. Notably, GNF-7 itself has since been recognized as a novel FLT3 inhibitor, capable of overcoming drug resistance in FLT3-ITD-positive acute myeloid leukemia.

### 4.2. Superposition of Imatinib’s Derivatives

**Figure 12:**
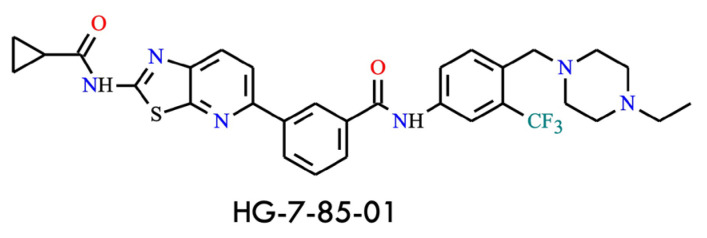
Structural formula of HG-7-85-01, a type II inhibitor active against BCR-ABLT^315l^ (The structure was drawn using ChemWindow 6.0 software).

The first example is HG-7-85-01 (Figure 12) [56], a hybrid compound combining structural elements of nilotinib and dasatinib. The guiding concept behind its design and synthesis was to integrate key pharmacophores into a single molecule to overcome resistance. Structurally, the aminothiazole moiety of dasatinib is fused to a pyridine ring, and this segment is connected to the phenyl-benzamide group of nilotinib, which confers selectivity toward the DFG-out conformation of BCR-ABL. This structural arrangement results in a cooperative inhibitor capable of effectively targeting the gatekeeper residue and showing activity against the BCR-ABL^T315I^ mutant in cellular assays (IC_50_ = 140 nM, less than threefold higher than for the wild type, IC_50_ = 58.5 nM) [3]. Furthermore, its selectivity profile is narrower than that of ponatinib in high-throughput screens, suggesting a potentially lower risk of adverse events-particularly cardiovascular toxicity-compared with ponatinib. Nevertheless, no clinical data or ongoing trials are currently available, making HG-7-85-01 an intriguing candidate for continued investigation [56].

**Figure 13:**
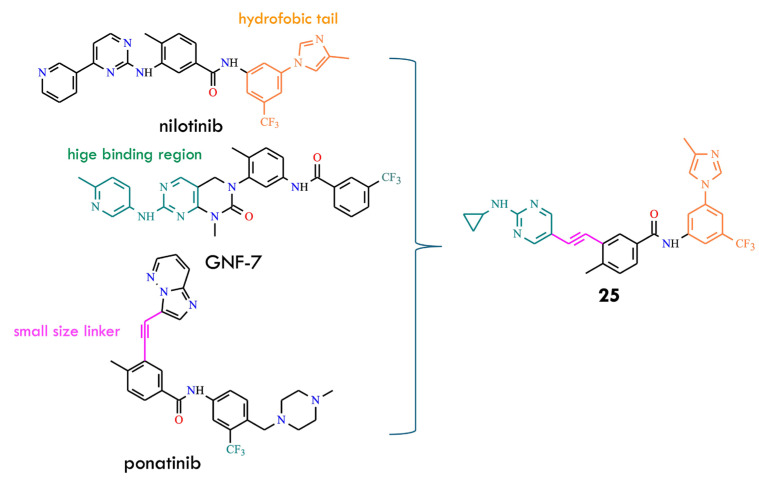
Design of BCR-ABL inhibitors **25** by hybridizing structural elements of nilotinib, GNF-7, and ponatinib (All structures were drawn using ChemWindow 6.0 software, based on data from ref. [57]).

Analysis of structural features reveals that nearly all approved type-II BCR-ABL inhibitors share three essential chemical components: a kinase hinge-binding moiety, a hydrophobic group interacting with the back pocket, and an appropriate linker connecting these two regions [57]. Guided by this rationale, the authors designed and synthesized new potential BCR-ABL inhibitors by hybridizing key structural elements derived from GNF-7, ponatinib, and nilotinib (Figure 13). The antineoplastic activity of the resulting hybrids was evaluated against BCR-ABL^T315I^, and one of the most potent compounds **25**, demonstrated an IC_50_ of 9.0 nM. Comparative antiproliferative assays on cancer cell lines showed that compound **25** displayed efficacy similar to ponatinib and GNF-7. Moreover, **25** induced dose-dependent G_0_/G_1_ cell-cycle arrest and apoptosis in K562 and Ba/F3 T315I cells. It also exhibited favorable pharmacokinetic properties and effectively suppressed tumor growth in xenograft mouse models bearing K562 or Ba/F3 cells expressing BCR-ABL^T315I^. These results indicate that **25** is a promising new antitumor candidate [57].

**Figure 14:**
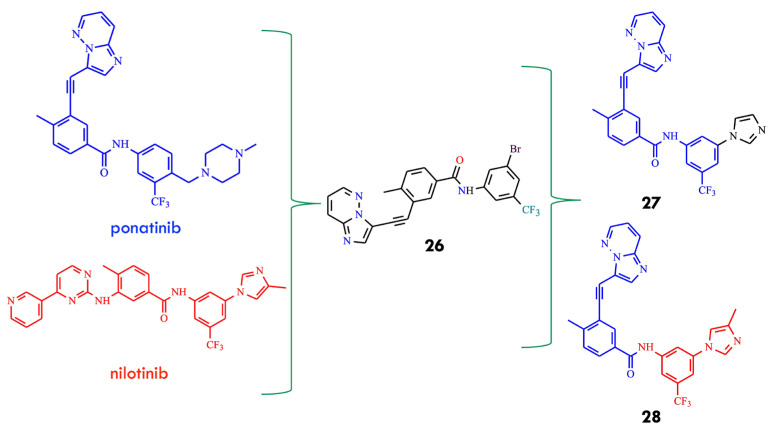
Design of the BCR-ABL inhibitors **27** and **28** using an intermediate structure **26** in a multi-track drug discovery approach (All structures were drawn using ChemWindow 6.0 software, based on data from ref. [58]).

Another design strategy has been aimed at mitigating ponatinib-associated cardiotoxicity, which is thought to arise from off-target inhibition of kinases essential for cardiovascular function due to its affinity for structurally similar ATP-binding pockets. The authors hypothesized that a TKI effective against both native and BCR-ABL^T315I^, while significantly safer for the heart than ponatinib, would broaden therapeutic options and alleviate clinical challenges for patients with CML and Ph^+^ ALL harboring T315I mutations. Hydrogen-bond interactions between TKIs and the Met318 residue in BCR-ABL are essential for inhibitory activity. Thus, re-engineering structural elements responsible for this interaction and exploring structure–activity relationships in terms of potency and cardiac safety could yield clinically superior compounds. The researchers first identified compound **26**, which displayed notable growth inhibition against native and mutant K-562 cells, with GI_50_ values of 18 and 370 nM, respectively [58]. Consistent with its cellular activity, compound **26** inhibited native BCR ABL and BCR-ABL^T315I^ kinases in biochemical assays with IC_50_ values of 150 and 360 nM, respectively, indicating its ability to reach the hydrophobic pocket of the T315I mutant. SAR analysis indicated that modifications at the R_1_ and R_2_ positions preserved key molecular recognition features. Substitution of the bromo group with imidazole or substituted imidazoles at the R_2_ position significantly enhanced inhibitory activity. Subsequent structural optimization led to the identification of inhibitors **27** and **28**, with mean IC_50_ values of 34.7 and 51.4 nM, respectively (Figure 14).

These analogs showed improved cardiac safety compared with ponatinib, inhibited BCR-ABL^T315I^ kinase activity, and suppressed proliferation of K-562 CML cells expressing the mutant kinase [58]. The discovery of compounds **27** and **28** was subsequently patented [59].

### 4.3. BCR-ABL Inhibitors Combined with Different Pharmacophores

**Figure 15:**
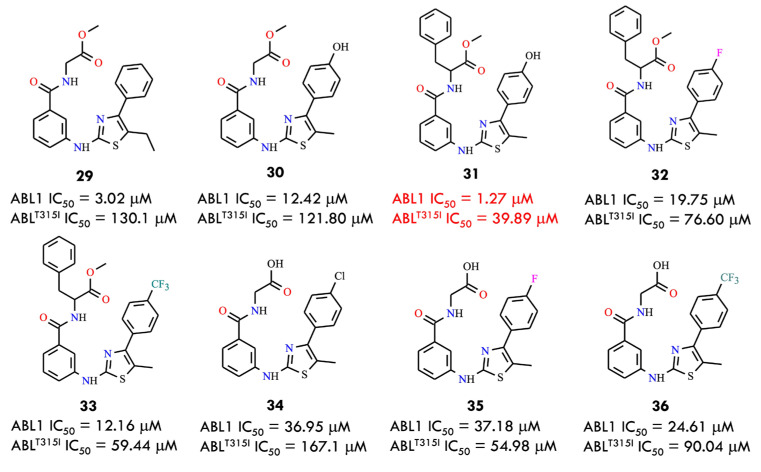
Structural formulas of thiazolamide-benzamide derivatives **29**–**36** and their IC_50_ values (All structures were drawn using ChemWindow 6.0 software).

Based on a comparative structural analysis of imatinib and ponatinib, the researchers proposed several design hypotheses. They speculated that introducing small-volume substituents in place of the pyrimidine ring of imatinib could reduce steric hindrance with the isoleucine residue in the mutant BCR-ABL kinase. To test this assumption, the 1,3-pyrimidine moiety was replaced with a 1,3-thiazole ring, while the benzamide pharmacophore, crucial for maintaining key interactions within the ATP-binding site, was retained. Using this strategy, the authors designed a series of novel thiazolamide–benzamide derivatives to improve the inhibitory profile against resistant BCR-ABL mutants. It was found that eight compounds (**29**–**36**) exhibited promising inhibitory activity (Figure 15)

Among them, the most potent derivative **31**, strongly inhibited BCR-ABL and BCR-ABL^T315I^, with IC_50_ values of 1.273 μM and 39.89 μM, respectively. In general, compounds conjugated with L-phenylalanine were more active than those linked to glycine, which may be attributed to the greater hydrophobicity of the benzyl group compared with hydrogen. This increased hydrophobic character likely enhances the inhibitory activity against both the wild-type and T315I mutant forms of the kinase. Notably, compound **31** represents a promising lead structure for the further development of broad-spectrum BCR-ABL inhibitors capable of overcoming clinically acquired resistance [1].

### 4.4. HDAC Inhibition as a Strategy to Overcome T315I-Mediated Resistance

**Figure 16:**
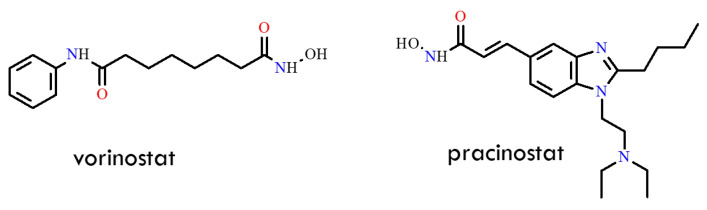
Structural formulas of the histone deacetylase inhibitors vorinostat, and pracinostat (All structures were drawn using ChemWindow 6.0 software).

Another promising strategy for overcoming the resistant T315I mutation was described in a 2013 study [60]. The authors focused on histone deacetylases (HDACs) and histone acetyltransferases (HATs), enzymes that regulate chromatin structure and function through acetylation and deacetylation processes essential for proper gene expression. In several tumor types, HDAC expression is upregulated, whereas HAT activity is dysregulated. HDAC inhibitors have therefore emerged as potent antitumor agents, capable of inducing cell-cycle arrest and apoptosis in malignant cells. Consequently, HDAC inhibition represents an appealing therapeutic approach in various malignancies. In the cited study, the activity of vorinostat and pracinostat was evaluated in combination with an Aurora kinase inhibitor. The authors [61,62] demonstrated that combining HDAC and Aurora kinase inhibitors markedly suppressed cell growth in BCR-ABL-expressing cells. Specifically, the Aurora kinase inhibitor tozasertib inhibited the proliferation of mutant BCR-ABL-expressing cells in a dose-dependent manner [63]. Co-treatment with HDAC inhibitors and tozasertib modulated intracellular signaling pathways in BCR-ABL-positive cells, and both vorinostat and pracinostat significantly reduced the growth of T315I-positive cells (Figure 16). Unfortunately, the combination index (CI) values for vorinostat–tozasertib and pracinostat–tozasertib were 0.396 and 0.765, respectively. These results indicate enhanced toxicity of these drug combinations in T315I-positive Ba/F3 cells. Nevertheless, the authors suggest that alternative dosing strategies or other Aurora/BCR-ABL dual inhibitors could potentially yield a more favorable therapeutic profile and offer clinical benefit to patients with CML.

**Figure 17:**
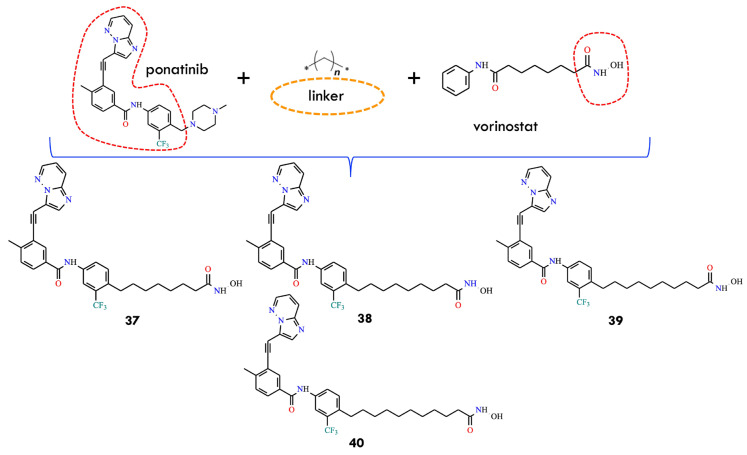
Key pharmacophores of ponatinib and vorinostat for constructing hybrid molecules **37**–**40** (All structures were drawn using ChemWindow 6.0 software, based on data from ref. [2]).

Another study investigated a combination of vorinostat, an HDAC inhibitor, with ponatinib, a multitargeted tyrosine kinase inhibitor. HDACs constitute a family of enzymes responsible for deacetylating lysine residues on histone and non-histone proteins [64]. The combined treatment of vorinostat and ponatinib significantly inhibited T315I-mutant cell growth compared with monotherapies. The enhanced efficacy suggests that dose reduction in both agents may be feasible, potentially reducing treatment-related adverse effects. In the most recent study discussed in this section, the authors designed a single hybrid molecule with dual inhibitory activity against both BCR-ABL and HDAC. For this purpose, the key pharmacophores of ponatinib and vorinostat were incorporated, and two types of biocompatible linker—methylene and 1,2,3-triazole-linked methylene—were used to connect the pharmacophores (Figure 17).

The inhibitory potential of the hybrid compounds was predicted using 2D-QSAR and 3D-pharmacophore models for BCR-ABL and HDAC. Molecular structures were prepared, and descriptors calculated using the Calculate Molecular Properties module in BIOVIA Discovery Studio [2]. Compounds showing docking scores below −30 kcal/mol in the active sites of BCR-ABL and HDAC were selected for further analysis. Hybrid molecules incorporating a methylene linker exhibited higher predicted binding affinity than those containing triazole-linked methylene. Notably, all methylene-linked hybrids (**37**, **38**, **39,** and **40**) demonstrated stronger binding than ponatinib to both wild-type and T315I-mutant BCR-ABL. Optimization of the methylene linker length led to the identification of the hybrid molecule **37**, featuring a linker composed of seven methylene units. Compound **37** exhibited potency comparable to ponatinib, but with an improved pharmacokinetic profile and reduced cardiotoxicity. The compound showed excellent predicted activity against BCR-ABL^T315I^, representing a promising direction for future anticancer drug development. However, further experimental validation is necessary to confirm its dual inhibitory activity against BCR-ABL and HDAC in vitro.

### 4.5. A Proteolysis Targeting Chimera (PROTAC)—Allosteric Inhibition Strategy of BCR-ABL Degraders

**Figure 18:**
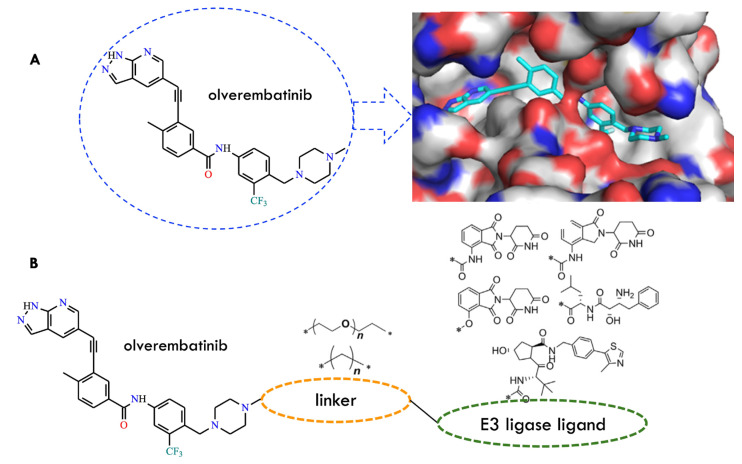
Design of BCR-ABL^T315I^ PROTACs. (**A**) Chemical structure of the BCR-ABL^T315I^ inhibitor olverembatinib and the proposed binding model of olverembatinib with the ABL1 protein. (**B**) General structure of the designed BCR-ABL^T315I^ PROTACs. (Reproduced from ref. [65], licensed under CC BY 4.0).

Patients with CML are exposed to the drawbacks of prolonged TKI administration, including cumulative toxicity and the emergence of resistance-conferring mutations. In this context, the PROTAC strategy has emerged as a novel protein-degradation technology capable of directly eliminating BCR-ABL, rather than merely inhibiting its kinase activity (Table 1) [65]. PROTACs (proteolysis-targeting chimeras) are heterobifunctional small molecules that induce selective degradation of a target protein by simultaneously engaging the protein of interest and an E3 ubiquitin ligase to form a ternary complex. PROTACs act catalytically and transiently, promoting ubiquitination and subsequent proteasomal destruction of the target protein. The first compound explored in this context was olverembatinib. Molecular docking studies demonstrated that it binds to the ATP-binding site in a type II manner, with its methylpiperazine moiety oriented toward the solvent region. This structural feature enabled its functionalization with an E3 ligase ligand for PROTAC design (Figure 18).

To generate BCR-ABL-directed PROTACs, three types of E3 ligases were evaluated: CRBN, VHL, and cIAP1, combined with two categories of linkers: ethylenedioxy linkers and aliphatic carbon chains. Additionally, a “hydrophobic tagging” strategy employing an adamantyl group was investigated; this approach induces misfolding and subsequent degradation of the target protein via the UPS [65].

**Figure 19:**
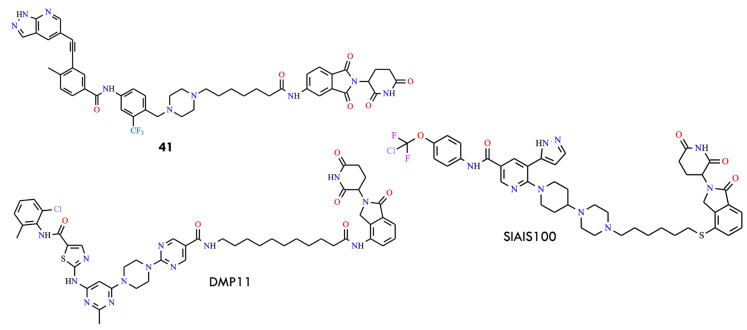
Structural formulas of the compound **41**, SIAIS100, and DMP11 (All structures were drawn using ChemWindow 6.0 software).

Among the synthesized derivatives, compound **41** (Figure 19), containing a six-carbon linker, displayed the most potent degradation activity, with an IC_50_ of 26.8 ± 9.7 nmol/L against Ba/F3 BCR-ABL^T315I^ cells. Mechanistic studies confirmed that degradation induced by **41** is mediated through the CRBN-dependent ubiquitin pathway. Importantly, compound **41** produced significant tumor regression in a Ba/F3 BCR-ABL^T315I^ xenograft model, suggesting its potential as a therapeutic candidate for overcoming the T315I mutation.

Another important advancement in the development of BCR-ABL PROTACs involves the identification of CRBN-based degrader SIAIS100 (DC_50_ = 2.7 nM), which effectively targets and degrades the BCR-ABL^T315I^ mutant by employing asciminib—an allosteric BCR-ABL inhibitor—as the recruiting ligand [32,66]. To improve solubility, the pyrrolidine-3-ol group at the asciminib linking site was replaced with a piperazine moiety. The researchers further developed compound DMP11, which degraded both BCR-ABL and SRC (Figure 19). This molecule was generated by incorporating a pyrimidine ring into the dasatinib scaffold and subsequently linking it to a CRBN ligand. DMP11 exhibited strong inhibitory activity against K562 and imatinib-resistant KA (K562) cells, with IC_50_ values of 0.261 nM and 0.837 nM, respectively.

### 4.6. BCR-ABL^T315I^ Inhibitors Incorporating Amino Acids as Flexible Linkers

**Figure 20:**
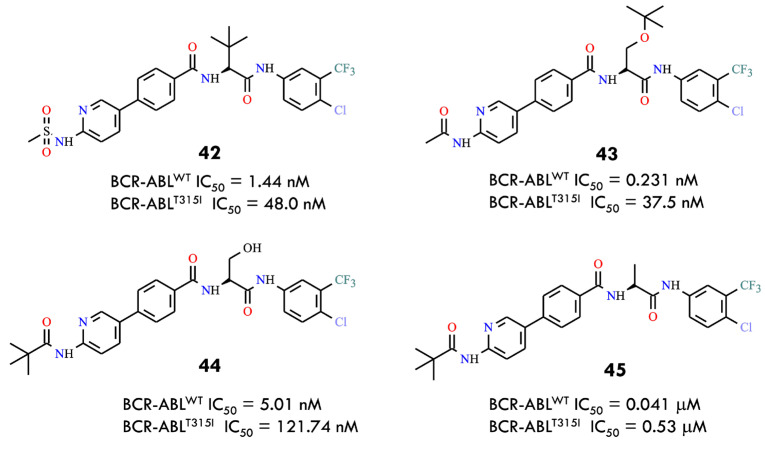
Structural formulas of the compounds **42**–**45** (All structures were drawn using ChemWindow 6.0 software).

Recent studies have reported a strategy employing amino acid-based flexible linkers to overcome resistance caused by the gatekeeper T315I mutation in BCR-ABL. Incorporation of an amino acid linker reduces steric clashes with the bulky side chain of Ile315, allowing the inhibitor to better access the ATP-binding pocket [67]. Simultaneously, modifications in the hinge-binding moiety (HBM) were introduced to restore the hydrogen-bond network disrupted by the T315I mutation, which is critical for stable inhibitor binding [68]. This approach enabled the design of novel BCR-ABL inhibitors with alternative chemical scaffolds. The amino acid linker improved not only binding affinity but also solubility, pharmacokinetic properties, and selectivity toward tumor cells, potentially reducing toxicity. A series of compounds was synthesized using amino acids such as serine and tert-leucine as flexible linkers. Several compounds, including **42**–**45**, demonstrated potent inhibitory activity against both BCR-ABL^WT^ and BCR-ABL^T315I^ and suppressed the proliferation of Ba/F3 cells expressing the T315I mutant (Figure 20). Some compounds also induced apoptosis, cell-cycle arrest, and inhibition of BCR-ABL and STAT5 phosphorylation. Molecular docking and in silico studies supported the rationale for amino acids as linkers: heterocyclic HBM structures connected via amino acids adopt favorable conformations, and phenylpyridine cores with amide side chains contribute to potent inhibitory activity. The authors concluded that the flexible amino acid linker strategy could serve as a general approach for the development of kinase inhibitors targeting proteins with bulky gatekeeper residues.

## 5. Second- and Third-Generation Inhibitors in the Context of the T315I Mutation

Resistance to imatinib led to the development of next-generation TKIs (Table 2). The T315I mutation—present in over 20% of patients with CML—results from the substitution of threonine with isoleucine at position 315 in the ABL1 kinase domain. The bulkier isoleucine residue blocks access to the ATP-binding pocket, preventing several inhibitors from forming critical interactions and thereby severely limiting their efficacy. BCR-ABL TKIs are categorized into mechanistic types based on their binding mode and conformational selectivity. Type I inhibitors bind the active conformation of the kinase and directly compete with ATP. Type II inhibitors recognize and stabilize the inactive DFG-out conformation, occupying both the ATP-binding cleft and an adjacent hydrophobic allosteric pocket. More recently, Type IV (allosteric) inhibitors have been developed; these compounds bind to regions spatially distant from the catalytic domain, inducing conformational changes that inhibit kinase activation.

Since its clinical introduction in 2001, imatinib has been the first-line therapy for CML, revolutionizing the treatment landscape. Imatinib binds the catalytic domain of ABL1 as a competitive ATP inhibitor, thereby preventing autophosphorylation of the BCR-ABL fusion protein and effectively suppressing the proliferation of Philadelphia chromosome-positive (Ph^+^) CML cells [67]. Subsequently, the second-generation TKIs nilotinib and dasatinib were developed to address imatinib resistance and are now widely used as front-line or second-line agents. Despite these achievements, resistance frequently emerges, particularly in advanced phases of CML. In addition, many ATP-competitive TKIs cause serious adverse effects, including increased vascular events with nilotinib, pulmonary hypertension and myelosuppression with dasatinib, and elevated hepatic enzymes (ALT, AST) with bosutinib. Although substantial efforts have been undertaken to develop TKIs active against T315I, many candidates were discontinued due to unacceptable toxicity. Interestingly, the ABL kinase shares extensive structural homology with cytoplasmic tyrosine kinases of the SRC family, which are commonly implicated in hematological and solid tumors. As a result, several compounds initially designed as SRC inhibitors were later found to inhibit BCR-ABL as well. Among these, the Type I dual BCR-ABL/SRC inhibitors dasatinib and bosutinib are prototypical representatives. Dasatinib, a substituted thiazole-carboxamide, is approved for first- and second-line CML therapy. Although highly potent, it remains ineffective against the T315I mutant. Compared with imatinib, dasatinib is structurally smaller and forms fewer fixed interactions with BCR-ABL.

NMR studies revealed that dasatinib variably binds BCR-ABL, accommodating both active and inactive conformations. The pyridine moiety present in imatinib is replaced by a hydroxyethyl-piperazine group, which remains solvent-exposed upon binding. Many conformation-altering mutations-except T315I-remain sensitive to dasatinib [3]. Bosutinib, a quinoline derivative, similarly binds BCR-ABL and c-SRC. Crystal structures show that the aniline moiety occupies a pocket adjacent to the ATP-binding site, oriented at ~65° relative to the quinoline plane. The quinoline nitrogen remains solvent-exposed, while the nitrile group forms a water-mediated hydrogen-bonding network that contributes to the kinase selectivity of bosutinib [69]. Radotinib (RAD, IY5511HCl; supect), a pyrazine–pyrimidine benzamide derivative [70], is a novel orally administered second-generation tyrosine kinase inhibitor with high affinity for BCR-ABL1 [71]. Structurally, it closely resembles imatinib and nilotinib. Developed to provide a more accessible therapeutic option in emerging regions, radotinib has been approved in South Korea for the treatment of chronic myeloid leukemia (CML). Phase II clinical trial data have demonstrated that radotinib is both efficacious and well-tolerated, yielding substantial major and complete cytogenetic response rates. However, compared with other second-generation TKIs such as nilotinib and dasatinib, radotinib exhibits a notable limitation: it lacks activity against the T315I BCR-ABL1 mutation. Even at the highest tested concentrations, radotinib failed to inhibit this mutation in clinical studies. Consistently, immunoblot analyses demonstrated that although radotinib effectively suppresses BCR-ABL kinase activity in wild-type and several mutant forms, it does not inhibit the kinase activity associated with the T315I variant [72]. Ponatinib (AP24534), a third-generation multikinase inhibitor, represented a breakthrough, as it exhibits potent activity against both native and T315I-mutant BCR-ABL. However, shortly after its approval, ponatinib was linked to serious vascular occlusive events-including coronary and cerebral thrombosis, myocardial infarction, and stroke-which led to temporary withdrawal from the market. Following reauthorization with revised indications and strengthened warnings, ponatinib remains available, but it is widely recognized as one of the most cardiotoxic TKIs. These limitations have intensified the search for therapeutic agents capable of targeting T315I with improved safety profiles.

**Figure 21:**
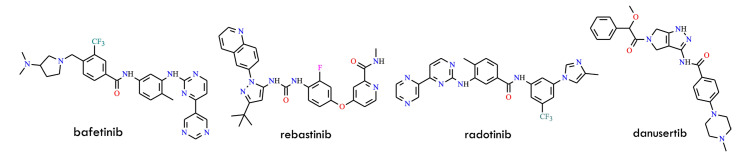
Structural formulas of the bafetinib, rebastinib, radotinib, and danusertib (All structures were drawn using ChemWindow 6.0 software).

Beyond approved agents, several additional inhibitors have been investigated for their ability to overcome TKI resistance (Figure 21). Bafetinib (INNO-406, NS-187), a dual/LYN inhibitor structurally related to imatinib, demonstrates activity against most kinase-domain mutants except T315I and is undergoing Phase I clinical evaluation. Rebastinib (DCC-2036), a Type II inhibitor, is capable of inhibiting T315I-mutant BCR-ABL and has shown sustained suppression of BCR-ABL signaling in early clinical trials. Danusertib (PHA-739358), initially characterized as an Aurora kinase inhibitor, also inhibits both wild-type and T315I-mutant BCR-ABL. Phase I studies have reported hematologic responses in refractory CML patients harboring T315I. Similarly, tozasertib-an aminopyrazole-pyrimidine derivative originally developed as a Type II Aurora kinase inhibitor-was later identified as a Type I BCR-ABL inhibitor. It binds the active kinase conformation with nanomolar potency against both native and T315I mutants. Clinical studies have demonstrated hematologic responses in approximately 45% of T315I-positive CML patients treated with tozasertib [3].

Asciminib was incorporated into the National Comprehensive Cancer Network (NCCN) guidelines as a new option for adults with Ph^+^ chronic-phase CML previously treated with ≥2 TKIs or carrying the T315I mutation [73]. This decision was supported by outcomes from the Phase 3 ASCEMBL trial (NCT03106779) and Phase 1 trial (NCT02081378). Asciminib’s unique mechanism—binding to the myristoyl pocket of BCR-ABL1 and locking the kinase in an inactive conformation—confers high specificity and selectivity, reducing off-target effects and enabling rapid, deep, and sustained molecular responses [74]. Currently, ponatinib is the only clinically approved third-generation tyrosine kinase inhibitor (TKI) for the treatment of CML, with regulatory approval granted by the FDA. Olverembatinib represents a newer agent targeting BCR-ABL, FLT3, KIT, and several other kinases. It was designed to inhibit both wild-type BCR-ABL and a broad spectrum of BCR-ABL mutants, including the T315I gatekeeper mutation [26]. Clinical studies of olverembatinib in CML are ongoing. Comparative studies indicate that olverembatinib exhibits cytostatic activity similar to ponatinib in imatinib-resistant cell lines [75]. Overall, significant efforts continue to focus on developing inhibitors capable of overcoming the T315I mutation, driven by the limitations of ATP-competitive TKIs and the unacceptable toxicity profiles that have halted the development of many earlier candidates.

## 6. Molecular Modeling Approaches for BCR-ABL^T315l^ Inhibitors

### 6.1. Comparative Molecular Docking Analysis of PBA2, CD-200 and JNJ- 26,854,165 with Ponatinib

**Figure 22:**
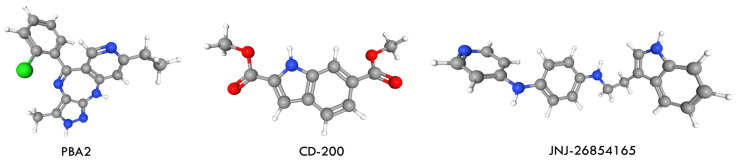
Ball and sticks three-dimensional structures of the BCR-ABL inhibitors PBA2, CD-200, and JNJ-26854165, obtained via molecular modeling (Structures were generated using Discovery Studio 2025 (BIOVIA)).

Ponatinib remains the only clinically approved inhibitor capable of effectively targeting the T315I-mutant BCR-ABL. Its temporary suspension in 2013 due to severe cardiovascular adverse events increased interest in alternative therapeutic strategies for CML. Several compounds-PBA2, CD-200, and JNJ-26854165 (JNJ-165, serdemetan) (Figure 22)-have since been reported to show inhibitory activity against T315I in both in vitro and in vivo studies [76].

The first approach involved combining low-dose ponatinib with other anticancer agents acting through distinct molecular pathways. PBA2 was selected because targeting BCR-ABL-independent pathways, such as Wnt/β-catenin signaling, represents a promising strategy. Wnt/β-catenin signaling is essential for hematopoietic stem cell self-renewal and differentiation, and its aberrant activation in CML promotes proliferation, survival, and TKI resistance. In CML cells, β-catenin preferentially recruits CBP over p300, supporting leukemic cell proliferation. CBP inhibition shifts β-catenin binding toward p300, thereby inducing differentiation and p53/p21-mediated senescence independently of BCR-ABL mutation status. PBA2 suppresses CBP expression, enhances β-catenin/p300 signaling, and demonstrates strong antiproliferative activity in both wild-type and T315I-mutant CML cells. It also reduces tumor volume and weight in CML models. These findings support PBA2 as a promising candidate for further investigation and as a potential strategy for overcoming TKI resistance in CML [77]. The second compound evaluated was CD-200, an extract enriched in sesquiterpene lactones from *L. tulipifera*. CD-200 inhibited the proliferation of both Ba/F3 WT and Ba/F3 T315I cells, blocked BCR-ABL signaling, and induced mitochondria-dependent apoptosis. It increased cytochrome c release, reduced MCL-1 and surviving expression, and promoted cleaved PARP accumulation, as well as the formation of TUNEL-positive apoptotic bodies. CD-200 also inhibited phosphorylation of BCR-ABL and its downstream targets STAT5 and CrkL in T315I-mutant cells, while imatinib did not. These data indicate that CD-200 overcomes imatinib resistance by effectively targeting the BCR-ABL pathway and inducing apoptosis, supporting its potential as a therapeutic option for CML with the T315I mutation [78]. The third compound, JNJ-26854165, is a tryptamine-derived MDM2 inhibitor with strong antiproliferative and cytotoxic effects in BCR-ABL-expressing cells, including primary blast-crisis samples and T315I-mutant cells. JNJ-165 induces cell death independently of p53 status, and its activity correlates with proteasome-dependent downregulation of BCR-ABL and inhibition of downstream signaling components such as CrkL and STAT3/5. Although combination therapy with imatinib or PD180970 results in synergistic effects in several CML models, JNJ-165 alone is highly effective against T315I-mutant cells, inducing near-complete regression of T315I-driven tumors and significantly prolonging survival in vivo. These findings suggest that JNJ-165, either as monotherapy or in combination with TKIs, is a promising strategy for overcoming TKI resistance in CML [79]. In this study, in silico approaches were used to investigate and compare the binding interactions, affinity, and inhibitory potential of these candidate molecules relative to ponatinib.

Molecular docking was performed using AutoDock Vina 4, employing a 40 × 40 × 40 Å grid box with a spacing of 0.357 Å. The best docking poses were selected based on binding energy and cluster size. Docking results revealed distinct interaction profiles for the candidate inhibitors:

- PBA2 exhibited a binding energy of −2.866 kcal/mol and formed hydrogen bonds, as well as π–cation and π–anion interactions with residues K271, E279, E282, E286, D381, and R386.
- CD-200 showed a docking score of −6.912 kcal/mol, establishing hydrogen bonds and π–π interactions primarily with residues M318 and F382.
- JNJ-26854165 demonstrated stronger affinity (−8.064 kcal/mol) through π–π interactions with Y253 and F382, and hydrogen bonds with E286 and N322.

In comparison, ponatinib displayed the most favorable docking score (−12.351 kcal/mol), forming multiple hydrogen bonds with key residues within the BCR-ABL^T315I^ binding pocket. Aromatic ring systems of all ligands were crucial for their binding interactions. Among the analyzed residues, Y253, K271, E279, E282, E286, M290, M318, N322, D381, F382, and R386 were identified as critical for ligand–receptor interactions. These results indicate that JNJ-26854165 exhibits higher binding affinity to BCR-ABL^T315I^ than PBA2 or CD-200, suggesting it as the most promising candidate among the three novel inhibitors. Nevertheless, ponatinib exhibited the greatest inhibitory potential, primarily due to its ability to form multiple stable hydrogen bonds within the mutant kinase domain. In summary, in silico analyses demonstrated that although PBA2, CD-200, and JNJ-26854165 can interact with the BCR-ABL^T315I^ active site, none match the binding efficiency or inhibitory potency of ponatinib. These findings highlight the importance of structural optimization and pharmacophore modeling based on potent inhibitors to guide the design of next-generation BCR-ABL inhibitors capable of overcoming the T315I mutation in CML [76].

### 6.2. Computer-Aided Design of Imatinib Derivatives

**Figure 23:**
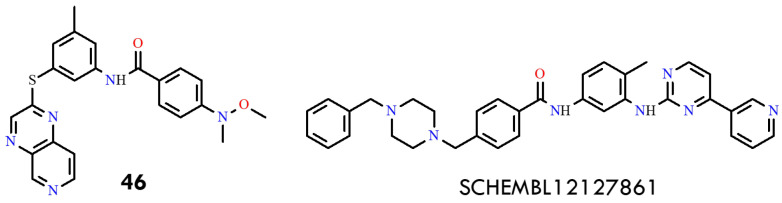
Structural formulas of compound **46** and SCHEMBL12127861 (All structures were drawn using ChemWindow 6.0 software).

This study employed a comprehensive in silico approach to design and characterize imatinib derivatives with improved affinity for the BCR-ABL^T315I^ kinase [80]. The crystal structure of the inactive (DFG-out) conformation of BCR-ABL (PDB ID: 6HD4; resolution 2.03 Å) was retrieved from the Protein Data Bank. Among the designed derivatives, compound **46** ([4-(methoxy(methyl)amino)-*N*-(3-methyl-5-(pyrido [3,4]pyrazin-2-ylthio)phenyl)benzamide]) and SCHEMBL12127861 ([4-((4-benzylpiperazin-1-yl)methyl)-*N*-(4-methyl-3-(4-(pyridin-3-yl)pyrimidin-2-ylamino)phenyl)benzamide]) (Figure 23) exhibited the most favorable binding energies (ΔG = −12.64 and −13.02 kcal/mol, respectively), comparable to imatinib (−12.62 kcal/mol) and superior to vodobatinib, radotinib, and flumatinib (Table 3).

Ponatinib, used as a reference, demonstrated ΔG values of −12.81 kcal/mol for mutant BCR-ABL. These results suggest that the newly proposed derivatives possess comparable or higher theoretical binding affinities relative to clinically utilized inhibitors. Docking studies were performed using AutoDock with the Lamarckian Genetic Algorithm (LGA). The receptor was treated as rigid, while the ligands were considered flexible. A grid box of 60 × 60 × 60 Å^3^ with 0.375 Å spacing was applied. Resulting protein-ligand complexes were analyzed using the Protein-Ligand Interaction Profiler (PLIP) to identify hydrogen bonds, π–π stacking, salt bridges, and hydrophobic contacts. Compound **46** exhibited an alternative binding orientation approximately 15 Å from the mutated gatekeeper site, engaging residues Pro230, Tyr232, Asp233, Glu236, Glu238, Asp241, and Lys263. Despite being outside the canonical ATP-binding cleft, these interactions may confer an advantage in overcoming steric resistance, representing a novel binding mode for BCR-ABL inhibition. SCHEMBL12127861 was positioned near the DFG motif, maintaining key contacts with Glu286, Val289, Leu298, Ala380, and Asp381. Asp381 formed a stable salt bridge and hydrogen bond with the piperazine nitrogen, similar to ponatinib-BCR-ABL^T315I^ complexes. Additional π–cation interactions between Arg386 and the 3-pyridyl ring, as well as van der Waals contacts with Glu286, Val289, Ile293, Ile360, and Phe382, further stabilized the complex. Compared with ponatinib, SCHEMBL12127861 displayed similar polar interaction profiles but greater hydrophobic complementarity near the activation loop, suggesting that hydrophobic forces substantially contribute to complex stabilization. Its flexible conformation allowed engagement with residues Asp363 and Arg386, interactions absent in ponatinib due to its rigid alkyne linker. These differences may explain the superior theoretical binding affinity of SCHEMBL12127861. Overall, SCHEMBL12127861 emerges as a promising lead compound for next-generation CML therapeutics. It is synthetically accessible and exhibits physicochemical properties comparable to imatinib, as supported by SwissADME predictions. While experimental validation remains necessary, these structural and energetic insights underscore its potential to overcome T315I-associated resistance [80].

### 6.3. Design, Synthesis, and Biological Evaluation of Novel 3-Amino-4-ethynyl Indazole Derivatives

**Figure 24:**
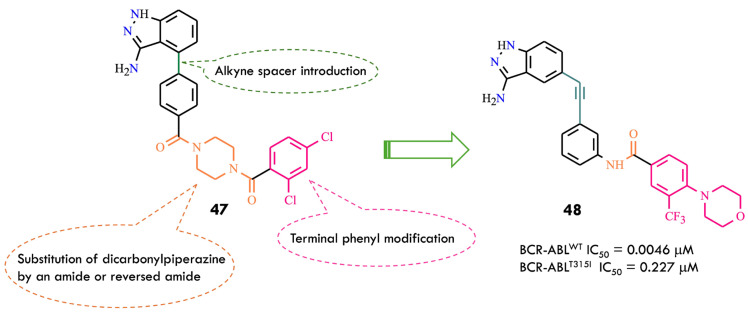
Rational design of compound **48** by structural modifications of **47** (All structures were drawn using ChemWindow 6.0 software, based on data from ref. [81]).

Ponatinib features an ethynyl linker connecting its hinge-binding motif (imidazo[1,2-b]pyridazine) to a diarylamide side chain. The slim acetylene spacer minimizes steric interference with the bulky isoleucine at the kinase gatekeeper region, while the terminal substituted 3-trifluoromethylphenyl group acts as a key allosteric binding moiety, optimizing BCR-ABL inhibitory activity. Structural modifications of ponatinib using a scaffold-hopping strategy led to equipotent analogs such as HQP1351 and a pyrazolopyrimidine derivative. These results indicate that connecting a hinge-binding headgroup to a diarylamide fragment via an alkyne bridge can yield novel chemical entities with potent BCR-ABL kinase inhibition. Among hinge-binding scaffolds, 3-aminoindazole is widely used in kinase inhibitor design. A series of aminoindazole-based BCR-ABL inhibitors were reported, among which compound **47** (Figure 24) exhibited modest enzymatic inhibition. Integration of key ponatinib structural domains into compound **47** aimed to enhance kinase inhibition and cellular potency.

A multidimensional structural optimization campaign was introduced: an ethynyl linker connecting indazole and diarylamide moieties; replacement of the dicarbonylpiperazine bridge with an amide or reversed amide linkage; modification of the terminal dichlorophenyl ring to substituted 3-trifluoromethylphenyl groups to improve enzymatic and cellular activity. Compound **48** emerged as a representative molecule, featuring: 3-aminoindazole hinge-binding motif forming hydrogen bonds with Met318 and Glu316; Ethynyl spacer bypassing steric hindrance from Ile315, enabling dual BCR-ABL^WT^ and BCR-ABL^T315I^ inhibition; 4-morpholino-3-trifluoromethylphenyl group engaging hydrophobic and polar interactions near the allosteric site. Molecular modeling showed canonical hydrogen bonds between the compound **48** amide linker and Glu286 (α-helix) and Asp381 (DFG-motif). The 3-aminoindazole NH and nitrogen atoms interacted with Glu316 and Met318, while the morpholino-trifluoromethylphenyl ring occupied the hydrophobic allosteric pocket. The ethynyl spacer facilitated van der Waals interactions with Ile315, avoiding steric clash. Docking indicated a Type II binding mode in the DFG-out conformation for both kinase forms [81].

### 6.4. In Silico Evaluation of 2,6,9-Trisubstituted Purine Derivatives as BCR-ABL^T315I^ Inhibitors

**Figure 25:**
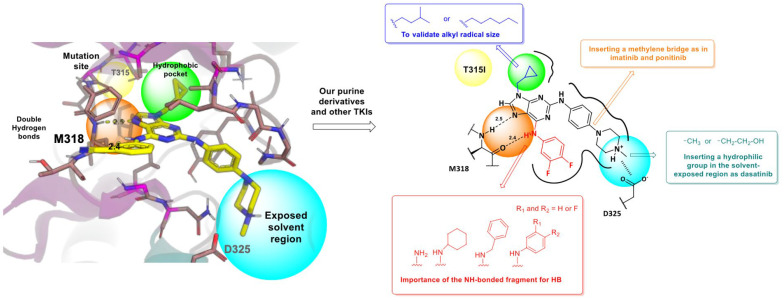
Design of new purine derivatives as potential BCR-ABL inhibitors. (Reproduced from ref. [82], licensed under CC BY 4.0).

The purpose of in silico studies is to validate hypotheses regarding the activity of novel compounds. In one study, the authors investigated a series of new BCR-ABL inhibitors based on a 2,6,9-trisubstituted purine scaffold. The three-dimensional structures of 14 final products were constructed using OECHEM, and conformers for these ligands were generated with OMEGA v.4.1.2.0 (Figure 25) [82].

The crystal structures of BCR-ABL^WT^ and BCR-ABL^T315I^ kinases were obtained from the RCSB Protein Data Bank (PDB IDs: 6BL8 and 4TWP, respectively). The hypothesis proposed that the antiproliferative effects of these purine derivatives in KCL22 B8 cells are related to both the presence of BCR-ABL in its T315I mutant form and the degree of inhibition conferred by the compounds. The results revealed a correlation between binding affinity energies and GI_50_ values, highlighting the positioning of the most potent antiproliferative compounds. Notably, structures containing an isopentyl group at the N9 position exhibited greater activity than those with a cyclopropylmethyl group, which corresponded with higher affinity for BCR-ABL^T315I^ [82].

**Figure 26:**
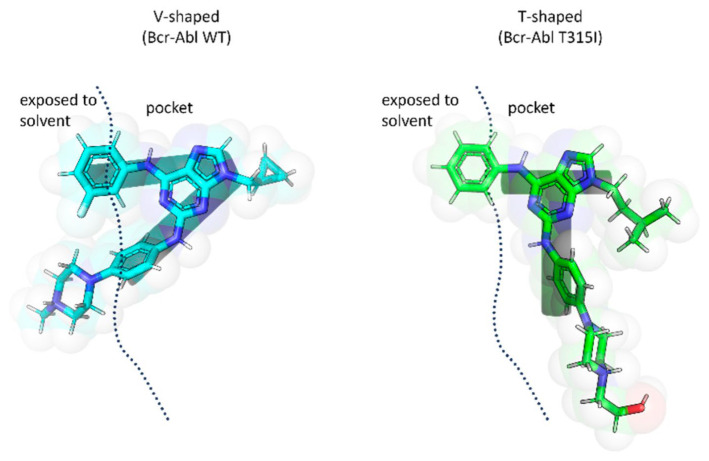
Predicted conformations of purine derivatives bound to BCR-ABL^WT^ and BCR-ABL^T315I^ (Reproduced from ref. [82], licensed under CC BY 4.0).

The study further demonstrated that the T315I mutation induces significant conformational changes in ligand binding. Ligands adopted a “V”-shaped conformation when bound to BCR-ABL^WT^, whereas a “T”-shaped conformation was preferred for BCR-ABL^T315I^ (Figure 26). This fit was facilitated by the larger hydrophobic region of the mutant kinase, which accommodated the N-9 isopentyl group, as well as by the optimal positioning of the phenylamino-N-hydroxyethyl-piperazine fragment at C2. These molecular docking results highlighted the key interactions of the inhibitors within the binding sites of both BCR-ABL^WT^ and BCR-ABL^T315I^ and explained the observed differences in potency. Additionally, the findings confirmed the relevance of specific substituents decorating the purine scaffold in modulating inhibitory activity.

### 6.5. 3D-QSAR Design of Purine-Based Antileukemic Derivatives Targeting BCR-ABL and the T315I Mutation

**Figure 27:**
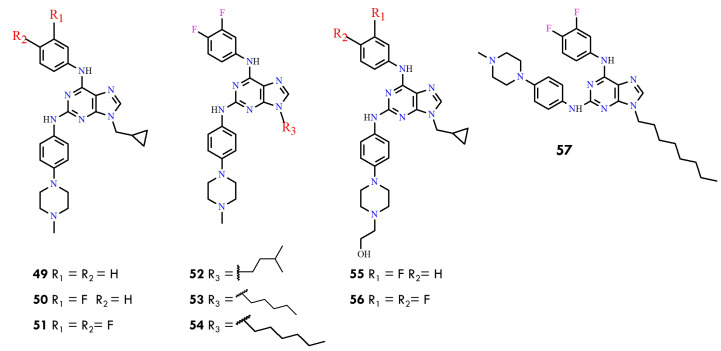
Structural formulas of purine derivatives **49**–**57** (All structures were drawn using ChemWindow 6.0 software).

Previous studies on purine derivatives demonstrated that compounds **49**–**51** (Figure 27) strongly inhibited BCR-ABL, with IC_50_ values ranging from 0.040 to 0.090 µM in the ABL kinase inhibition assay [83]. Among them, compound **51** showed the highest selectivity for BCR-ABL over other tyrosine kinases. It also exhibited low micromolar cytotoxicity in multiple leukemia cell lines and effectively reduced phosphorylation of downstream components of the BCR-ABL signaling pathway. These studies further identified the cyclopropylmethyl substituent at N9-present in compounds **49**–**51** as the optimal group for activity. This conclusion was supported by the markedly reduced potency of derivatives **52**–**54**, which contain longer hydrophobic chains at N9, and is consistent with docking results indicating steric limitations within the hydrophobic pocket of BCR-ABL.

The structure–activity relationships toward the BCR-ABL^T315I^ mutant were subsequently evaluated using compounds **55** and **56**. Compound **56** showed limited utility due to its weaker inhibitory profile, whereas compound **55** emerged as the most potent inhibitor, demonstrating strong activity against both wild-type and T315I-mutant BCR-ABL. Importantly, compound **55** was able to overcome resistance associated with the T315I gatekeeper mutation. Its potency and favorable inhibitory characteristics provided the foundation for further structural optimization in the present work. In this study, new purine derivatives were rationally designed as BCR-ABL inhibitors using 3D-QSAR models, which guided modifications at the C2, C6, and N9 positions of the purine scaffold. The 3D-QSAR analysis highlighted several key structural features required for optimal activity:

- The methylcyclopropyl group at N9 is markedly superior to longer hydrophobic chains such as n-hexyl, as evidenced by the weak activity of compound **57** (IC_50_ = 86.46 µM);
- The hydroxymethyl substituent on the piperazine ring should be retained, as seen in the highly active compounds **55** and **56**, in agreement with CoMSIA maps indicating that hydrophilic groups enhance BCR-ABL inhibition;
- CoMSIA results also showed that incorporation of an electronegative atom in the C2 phenylamino fragment favors activity;
- Contour maps suggested that electron-rich or electronegative groups in red regions and electron-deficient groups in blue regions improve inhibitory potency.

Accordingly, future modifications at the C6 phenylamino ring—particularly replacing meta- or para-fluorine atoms with substituents of varied steric and electronic properties—may help elucidate the optimal substitution pattern at this position, which remains unresolved.

**Figure 28:**
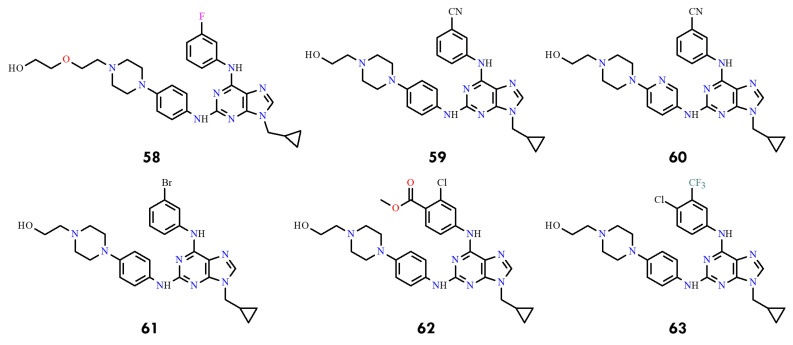
Structural formulas of purine derivatives **58**–**63** (All structures were drawn using ChemWindow 6.0 software).

Guided by these insights, seven new 2,6,9-trisubstituted purine derivatives were synthesized and evaluated, designated as compounds **58**–**63** (Figure 28). Among them, compounds **58** and **60** displayed the strongest inhibitory activity against BCR-ABL (IC_50_ = 0.13 and 0.19 µM, respectively), surpassing imatinib (IC_50_ = 0.33 µM). Compound **60** also exhibited potent cytotoxicity against imatinib-sensitive K562 and KCL22 cells while showing reduced toxicity toward non-neoplastic HEK293T cells, highlighting its potential as a promising lead compound.

**Figure 29:**
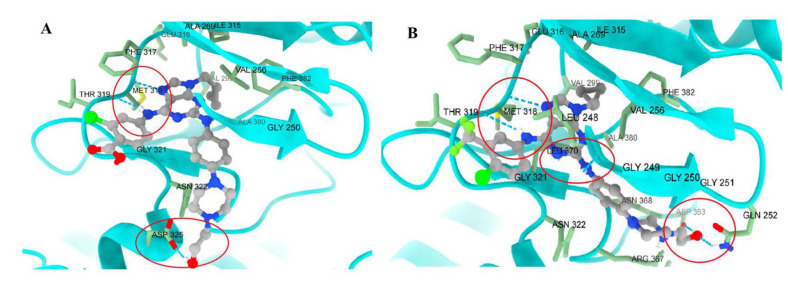
Comparison of poses of **62** (**A**) in BCR-ABL^T315I^ and **63** (**B**) in BCR-ABL^T315I^. In both cases, the ligands remain largely unaffected by the T315I mutation, suggesting stable docking in the presence of this mutation. Additionally, each ligand forms a new hydrogen bond: **63** interacts with GLN252, LEU248, and ASP353, while **62** interacts with ASP325. The most important interactions are highlighted in red circles (Reproduced from ref. [83], licensed under CC BY 4.0).

The most notable findings were observed in the imatinib-resistant context. KCL22-B8 cells expressing BCR-ABL^T315I^ exhibited markedly greater sensitivity to compounds **62** and **63** than to imatinib. Molecular docking supported these results, as both compounds displayed favorable binding energies (–10.186 and –10.457 kcal/mol). Moreover, molecular dynamics simulations revealed stabilizing interactions involving the hydroxyethyl fragment, suggesting that **62** (Figure 29A) and **63** (Figure 29B) adopt a binding mode particularly well suited to the T315I mutant.

Collectively, these findings indicate that although **58** and **60** are the most potent inhibitors of wild-type BCR-ABL, compounds **62** and **63** represent promising leads for overcoming T315I-mediated resistance. Structure–activity relationships further indicated that replacing the benzene ring with a pyridine moiety at C2 improves activity (**60** vs. **59**), whereas enlarging substituents at C6 or replacing fluorine with bulkier groups generally reduced potency.

Overall, this work demonstrates that integrating rational design with 3D-QSAR, molecular docking, and molecular dynamics simulations provides an effective strategy for developing next-generation purine-based BCR-ABL inhibitors. The newly synthesized derivatives not only outperform imatinib against the native kinase but also exhibit selective activity against the clinically challenging T315I mutant. Additionally, pharmacokinetic predictions indicate that several candidates, including **59** and **60**, may possess favorable properties for oral administration. These findings underscore the potential of purine scaffolds as strong starting points for future CML therapies, including treatments aimed at drug-resistant variants of the disease.

### 6.6. Spatial Interaction Analysis of Lead Compounds

**Figure 30:**
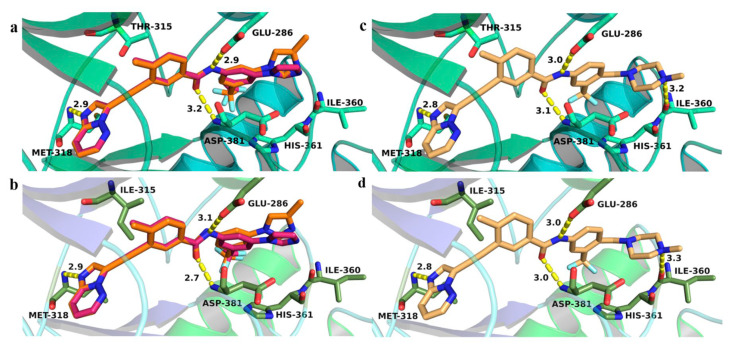
Potential binding modes of ponatinib and lead inhibitors **27** and **28** with BCR-ABL^WT^ (PDB ID: 3OXZ) and BCR-ABL^T315I^ (PDB ID: 3IK3). (**a**,**b**) The binding interactions of inhibitors **27** and **28**, respectively. (**c**,**d**) The binding interactions of ponatinib. Key residues that potentially form critical interactions with the inhibitors are shown in stick representation and labeled. Distances between interacting atoms are indicated by yellow dashed lines and labeled in black (Reproduced from ref. [58], licensed under CC BY-NC-ND 4.0).

In a recent study, most of the newly designed molecules were observed to form key hydrogen bond interactions with the backbone of Met318 in the hinge region of native BCR-ABL. Hybrids based on the ponatinib scaffold occupied the ATP-binding pocket of BCR-ABL^T315I^ and were predicted to form a hydrogen bond with the backbone of the methionine residue (Met318). As illustrated in Figure 30, lead compounds **27** and **28** occupied the same binding region in BCR-ABL^T315I^ as ponatinib and maintained similar distances between the N atom of Met318 and the N atom of the imidazo[1,2-b]pyridazine moiety of the inhibitors.

The spatial orientation relative to other key residues, such as Glu286 and Asp381, was comparable to that observed for ponatinib. Superimposition of BCR-ABL^WT^ and BCR-ABL^T315I^ with these lead compounds indicated that the ethynyl linker in the inhibitors bypassed the mutated gatekeeper residue Ile315 like ponatinib [58].

**Figure 31:**
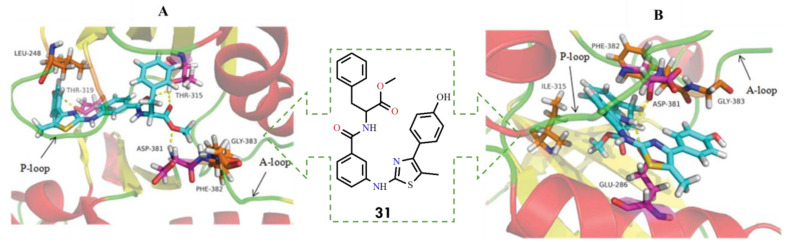
Chemical structure of **31** and the predicted binding mode of **31** with BCR-ABL and BCR-ABL^T315I^. (**A**) The binding mode of **31** with the ATP-binding site of BCR-ABL. Compound **31** is shown in blue with a translucent molecular surface. Hydrogen bonds are highlighted with yellow dashed lines. The residues forming hydrogen bonds with **31** are shown in purple. (**B**) The binding mode of **31** with the ATP-binding site of BCR-ABL^T315I^. The side chain of the mutated gatekeeper residue Ile315 is shown in purple (Reproduced from ref. [1] © 2019 The Royal Society of Chemistry, RSC Advances).

Another study employed molecular docking to predict the binding affinity of newly designed compounds. Docking simulations were performed using SYBYL-X2.0, with T315I (PDB code: 3IK3) as the receptor. Ligands were constructed in ChemDraw Ultra 7.0 and energy-minimized under the Tripos Force Field with Gasteiger–Hückel charges. Among the tested molecules, compound **31** displayed strong interactions with the gatekeeper residue Thr315, forming two hydrogen bonds. The terminal methoxyl group occupied a hydrophobic pocket near Asp38, where it established additional stabilizing hydrogen bonds, while the hydroxyl group on its benzene ring formed another hydrogen bond with Thr319. Overall, the docking results indicated that **31** fit well within the ATP-binding site without including steric hindrance with Ile315 (Figure 31) [1].

To complement these findings, the binding mode of HQP1351 was investigated computationally for both wild-type and T315I-mutant BCR-ABL. The compound bound tightly to the ATP-binding site of nonphosphorylated (DFG-out) BCR-ABL^WT^ and BCR-ABL^T315I^. Its 1*H*-pyrazolo[3,4-b]pyridine core occupied the adenine pocket and formed a hydrogen-bond network with the backbone of M318 in the hinge region. Two additional hydrogen bonds were provided by the amide moiety interacting with E286 and D381, whereas the trifluoromethylphenyl group engaged the hydrophobic pocket. Importantly, the alkynyl linker of HQP1351 enabled favorable van der Waals contacts with the I315 gatekeeper residue in BCR-ABL^T315I^ without generating steric clashes. The methylpiperazine group may further contribute through hydrogen bonding with I360 or H361 [14]. Further analysis showed that HQP1351 could also bind to phosphorylated BCR-ABL in its DFG-in conformation. In this state, pronounced structural differences in the activation loop relative to the DFG-out conformation resulted in a markedly altered binding mode: a substantial portion of HQP1351 extended beyond the ATP-binding pocket toward solvent-exposed regions of the protein.

### 6.7. Early Drug Discovery Concept of Fungal Metabolites Against the BCR-ABL^T315I^ Gatekeeper Mutation

**Figure 32:**
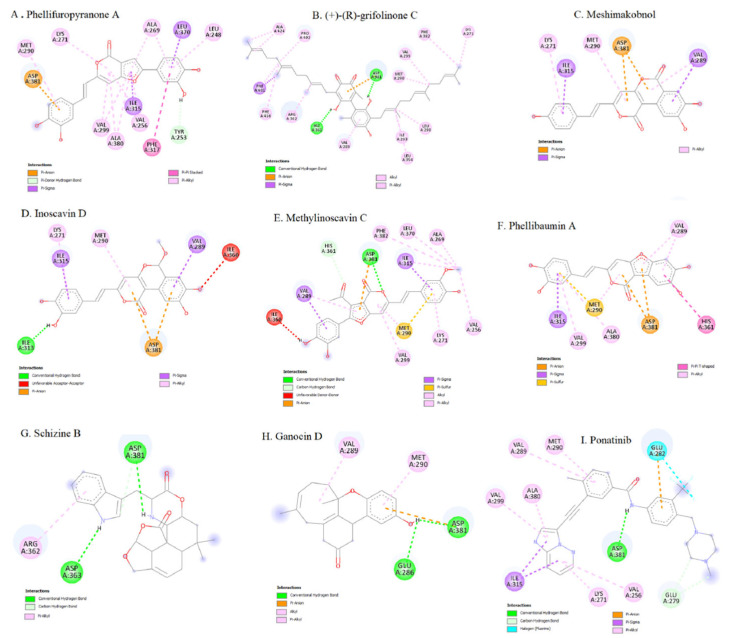
The 2-dimensional interactions of eight drug-like fungal secondary metabolites with the amino acid residues of T315I-BCR:ABL1 mutant protein (Reproduced from ref. [84], licensed under CC BY 4.0).

The persistent challenge posed by the BCR-ABL^T315I^ gatekeeper mutation has prompted a search for structurally novel therapeutic agents capable of overcoming resistance to current TKIs. In this context, fungal secondary metabolites have emerged as an intriguing and increasingly investigated source of potential inhibitors [84]. Fungi are known to produce an exceptionally wide array of chemically diverse natural products, including polyketides, terpenoids, alkaloids, and phenolic compounds, many of which display potent bioactivities across various molecular targets. This evolutionary diversity makes fungal metabolites particularly valuable in early-stage drug discovery, especially when targeting mutated kinases with sterically constrained binding pockets such as BCR-ABL^T315I^. Although research in this area is still at a preliminary stage-requiring synthetic confirmation, biochemical evaluation, and subsequent in vitro and in vivo validation-computational screening efforts have provided promising early insights. Recent molecular docking analyses revealed a broad range of predicted binding affinities across fungal metabolites screened against the T315I-mutated kinase. Notably, 27 metabolites exhibited higher predicted binding affinities than the reference inhibitor used in the study, highlighting the potential of natural-product scaffolds to engage the altered ATP-binding site created by the T315I substitution. Among these candidates, eight metabolites stood out not only for their docking performance but also their favorable drug-likeness profiles, suggesting a realistic potential for progression into lead-optimization workflows. These include Phellifuropyranone A (MSID001271), (+)-(*R*)-Grifolinone C (MSID000080), Meshimakobnol B (MSID001179), Inoscavin D (MSID001082), Methylinoscavin C (MSID001200), Phellibaumin A (MSID001266), Schizine B (MSID001385), and Ganocin D (MSID000814) (Figure 32). Many of these compounds belong to structural classes associated with antioxidant, antimicrobial, or cytotoxic activities-functional attributes that often correlate with the capacity to interact with kinase signaling pathways. Importantly, fungal metabolites may offer several advantages over purely synthetic molecules. Their structurally complex scaffolds introduce unique three-dimensional architectures and reactive functionalities that are often difficult to design de novo yet may be essential for effective interaction with the sterically hindered T315I mutant site. Natural products also frequently serve as valuable starting points for semi-synthetic optimization, allowing refinement of potency, selectivity, and pharmacokinetic properties. This is particularly relevant for the T315I mutation, where successful inhibition requires accommodating both the increased steric bulk and the altered hydrogen-bonding environment caused by the threonine-to-isoleucine substitution. Two-dimensional interaction analyses of the T315I-BCR:ABL1 mutant with eight drug-like fungal metabolites revealed that these compounds engage several key regulatory residues within the kinase domain. Among these metabolites, Phellifuropyranone A, (+)-(*R*)-grifolinone C, and Meshimakobnol B were prioritized for molecular dynamics (MD) simulations based on their binding affinities and favorable drug-likeness parameters. Notably, Meshimakobnol B formed the highest number of hydrogen bonds (four) with the mutant protein (Figure 32).

Interaction analyses of the T315I-BCR-ABL1 mutant with eight drug-like fungal metabolites showed that several compounds engage key regulatory residues within the kinase domain. Phellifuropyranone A, (+)-(*R*)-grifolinone C, and Meshimakobnol B were selected for molecular dynamics (MD) studies based on their binding affinities and drug-likeness features. Meshimakobnol B formed the largest number of hydrogen bonds with the mutant protein, and in 100 ns MD simulations, complexes with Meshimakobnol B and Phellifuropyranone A demonstrated superior stability compared with the protein–ponatinib complex, which displayed increased conformational fluctuations. Overall, Phellifuropyranone A, (+)-(*R*)-grifolinone C, and Meshimakobnol B exhibited more stable and/or more favorable interactions with the T315I mutant than ponatinib. Binding free-energy calculations further supported these results, with Phellifuropyranone A and Meshimakobnol B displaying energy values comparable to ponatinib, driven largely by favorable van der Waals and hydrogen-bonding contributions. Together, these findings highlight fungal secondary metabolites as promising and underexplored candidates for T315I-BCR-ABL1 inhibition. Their early in silico performance supports the need for further evaluation through comprehensive in vitro and in vivo studies, toxicity assessments, formulation optimization, and off-target profiling to advance them toward potential therapeutic development for CML.

## 7. Perspectives and Future Directions

As demonstrated in previous sections, numerous chemical scaffolds show considerable promise for the future treatment of chronic myeloid leukemia (CML), highlighting the ongoing need for innovative therapeutic strategies to overcome drug resistance. The structural diversity of these compounds enables multiple approaches to overcoming resistance mechanisms, including multitarget inhibition and rational scaffold modification. Early studies indicated that optimized combinations of HDAC inhibitors with Aurora kinase inhibitors may reduce toxicity while maintaining therapeutic efficacy, while dual Aurora/BCR-ABL inhibitors could offer advantages over single-target BCR-ABL inhibitors. Compounds structurally related to GNF-7 remain underexplored and represent a valuable direction for future investigation. HG-7-85-01 is particularly noteworthy due to its distinctive structural features and improved safety profile, including reduced cardiovascular toxicity compared with ponatinib. Similarly, compounds **27** and **28** were designed to mitigate ponatinib-associated cardiotoxicity, reflecting a broader trend toward safer next-generation inhibitors.

The composite compound HS-438, which shares structural motifs with sorafenib and regorafenib and targets VEGF and PDGFR-β, represents another promising scaffold. Given that *N*-methylpicolinamide derivatives have not yet demonstrated activity against BCR-ABL^T315I^, HS-438 may provide a useful starting point for further optimization. Its 2-benzothiazolyl urea core has inspired related derivatives, including compounds **18**, **20**, and **21**, with structural modifications—such as replacement of the oxypicolinamide group with a 2-methoxyphenyl substituent in **21**—broadening anticancer activity. Clinically approved drugs such as imatinib and ponatinib continue to serve as valuable templates for structural modification. For example, compound **31**, in which the 1,3-pyrimidine ring was replaced by a 1,3-thiazole moiety, represents a promising lead. In addition, 2,6,9-trisubstituted purines and natural products derived from fungal sources offer chemically diverse frameworks for inhibitor development. Molecular docking studies across multiple compound classes revealed favorable interactions with key regulatory residues of the BCR-ABL^T315I^ kinase domain, including hydrogen bonding to the methionine backbone, a feature considered critical for inhibitory activity. Future research should incorporate comprehensive in vitro kinase inhibition assays, pharmacophore modeling, and rational optimization strategies to improve selectivity while minimizing off-target effects. Collectively, these approaches may facilitate the development of potent and safer BCR-ABL^T315I^ inhibitors.

## 8. Conclusions

In this review, we summarized the diverse medicinal chemistry strategies currently being pursued to overcome the BCR-ABL^T315I^ gatekeeper mutation, one of the most challenging resistance mechanisms in chronic myeloid leukemia therapy. Approaches aimed at alleviating steric hindrance introduced by the Ile315 substitution include rational modifications of imatinib-derived scaffolds as well as the repurposing and optimization of inhibitors originally developed for other kinase targets, such as Aurora kinases, which were successfully adapted to the ABL active site. Combination and hybrid strategies, particularly those involving HDAC inhibitors and multitargeted kinase inhibitors, have emerged as promising avenues for improving therapeutic efficacy while reducing toxicity through dose optimization. In parallel, a wide range of chemically diverse scaffolds—including heterocyclic systems, urea-based hybrids, and modified kinase inhibitor backbones—have demonstrated encouraging activity against the T315I mutant, with recent efforts increasingly focused on achieving balanced efficacy, improved safety profiles, and favorable pharmacokinetic properties. Notably, hybrid molecules inspired by clinically established inhibitors such as ponatinib, nilotinib, and dasatinib illustrate how rational scaffold re-engineering can address resistance while mitigating adverse effects, including cardiovascular toxicity. Finally, molecular modeling and simulation techniques continue to play a critical role in elucidating ligand–target interactions, guiding structure-based optimization, and supporting the development of next-generation BCR-ABL^T315I^ inhibitors.

## Figures and Tables

**Table 1:** Comparison of selected BCR-ABL PROTAC degraders.

Compound	BCR-ABL Ligand	Inhibitor Type	E3 Ligase Ligand	Linker/ Modification	Degraded Target(s)	Key Parameters	Major Biological Findings
**41**	HQP1351 derivative	ATP-competitive, type II	CRBN	Six-carbon aliphatic linker	BCR-ABL^T315I^	IC_50_ = 26.8 ± 9.7 nM (Ba/F3 BCR-ABL^T315I^)	Significant tumor regression in Ba/F3 BCR-ABL^T315I^ xenograft model; degradation mediated by CRBN pathway
SIAIS100	asciminib	Allosteric inhibitor (myristate pocket)	CRBN	Replacement of pyrrolidine-3-ol with piperazine (increased solubility)	BCR-ABL^T315I^	DC_50_ = 2.7 nM	Potent degradation of BCR-ABL^T315I^; optimized CRBN-based degrader reported in 2025
DMP11	Modified dasatinib (pyrimidine-ring introduction)	ATP-competitive	CRBN	Dasatinib–pyrimidine scaffold linked to CRBN ligand	BCR-ABL, SRC	IC_50_ = 0.261 nM (K562), 0.837 nM (KA, imatinib-resistant K562)	Efficient degradation of BCR-ABL and SRC; high activity in sensitive and imatinib-resistant cells

**Table 2:** Overview of selected BCR-ABL inhibitors and their activity against the T315I mutation.

Inhibitor	Generation	Binding Type	Activity vs. T315I	Main Advantages	Main Limitations (Toxicity/Resistance)	Clinical Status
ponatinib (AP24534)	III	Type II (DFG-out), ATP-competitive	High/full activity	Potent inhibitor of T315I and most K_D_ mutations; effective in CML and Ph^+^ ALL	High risk of vascular events (thrombosis, stroke, myocardial infarction), cardiotoxicity	Approved (FDA 2012)
olverembatinib (HQP1351)	III	Type II (DFG-out), ATP-competitive	High/full activity	Potent inhibitor of T315I, binds to both the phosphorylated and non-phosphorylated forms of BCR-ABL, also to, e.g., KIT, PDGFRα kinases.	Thrombocytopenia, anemia, and neutropenia, skin pigmentation, and elevated levels of creatinine kinase.	Approved (NMPA 2021)
asciminib (ABL001)	allosteric	Type IV (myristoyl pocket binder)	High, including against T315I	Highly selective; reduced off target toxicity; synergistic with ATP-competitive TKIs	Resistance via myristoyl-pocket mutations (e.g., A337V, P465S)	Approved (FDA 2021)
rebastinib (DCC-2036)	II/next-generation	Type II stabilizes DFG-out	Demonstrated activity in clinical studies	Inhibits T315I and multiple mutations; durable signaling suppression	Limited clinical data; not approved	Clinical trials (Phase I/II)
danusertib (PHA-739358)	–	Aurora kinase inhibitor with off-target BCR–ABL inhibition	Activity confirmed in vitro and clinically	Inhibits T315I; hematologic responses observed	Hematologic toxicity; limited selectivity	Phase I completed
tozasertib (MK-0457, VX-680)	–	Type I (active-state binder)	Strong activity against T315I	Nanomolar potency; ~45% clinical response rate	QT prolongation, myelosuppression; program discontinued	Clinical development terminated
bafetinib (INNO-406, NS-187)	II	Type II	No activity against T315I	ABL/LYN inhibitor; active against several other mutations	Ineffective against T315I	Phase I
bosutinib (Bosulif)	II	Type I	No activity against T315I	Broad kinase selectivity	Ineffective against T315I	Approved (FDA 2012; EMA 2013)
dasatinib (Sprycel)	II	Type I/dual SRC–ABL	No activity against T315I	High potency; effective against many non-T315I mutations	Ineffective against T315I	Approved (FDA 2006; EMA 2006)
radotinib (RAD, IY5511HCl; supect)	II	ATP-competitive	No activity against T315I		Ineffective against T315I	Phase III

**Table 3:** Comparative analysis of BCR-ABL^T315I^ inhibitors.

Inhibitor	Generation/Type	Docking Score (kcal/mol)	Key Interactions	Binding Mode	Notes/Advantages
ponatinib	third generation, Type II	−12.351	H-bonds: Y253, K271, E279, E282, E286, M290, M318, N322, D381, F382, R386	DFG-out (Type II)	Clinically approved; most potent; forms multiple stable H-bonds; overcomes T315I; cardiotoxicity concerns
PBA2	novel candidate	−2.866	H-bonds, π–cation, π–anion: K271, E279, E282, E286, D381, R386	ATP-binding site	Moderate binding; lower affinity than JNJ-26854165; early-stage research
CD-200	novel candidate	−6.912	H-bonds, π–π: M318, F382	ATP-binding site	Higher affinity than PBA2; still lower than JNJ-26854165; preclinical stage
JNJ-26854165 (JNJ-165, serdemetan)	novel candidate	−8.064	H-bonds: E286, N322; π–π: Y253, F382	ATP-binding site	Most promising among new candidates; strong binding interactions; preclinical
**46**	imatinib derivative	−12.64	H-bonds: Pro230, Tyr232, Asp233, Glu236, Glu238, Asp241, Lys263	Alternative site (~15 Å from gatekeeper)	Novel binding orientation; may overcome steric hindrance from T315I
SCHEMBL12127861	imatinib derivative	−13.02	H-bonds: Glu286, Asp381; π–cation: Arg386; van der Waals: Glu286, Val289, Ile293, Ile360, Phe382	Near DFG motif; Type II-like	High theoretical binding; strong hydrophobic stabilization; flexible to engage additional residues; promising lead compound
**48** (3-amino-4-ethynyl indazole)	ponatinib-based derivative	–	H-bonds: Glu286, Asp381, Met318, Glu316; van der Waals: Ile315	DFG-out (Type II)	Dual BCR-ABL^WT^ and T315I inhibition; ethynyl linker bypasses steric hindrance; strong polar and hydrophobic interactions; experimental validation ongoing
olverembatinib/ pyrazolopyrimidine derivatives	ponatinib derivative	–	–	Type II	Alternative hinge-binding scaffolds; retains ethynyl bridge and diarylamide fragment; potent BCR-ABL inhibition

## Data Availability

No new data were created or analyzed in this study. Data sharing is not applicable to this article.

## Abbreviations

The following abbreviations are used in this manuscript:

ABL	Abelson kinase
ALT	Alanine aminotransferase
AML	Acute Myeloid Leukemia
AST	Aspartate aminotransferase
Ba/F3	Murine pro-B cell line (interleukin-3 dependent)
BCR	Breakpoint Cluster Region
CAR-T	Chimeric antigen receptor T-cell therapy
CBP	CREB-binding protein
cIAP1	Cellular Inhibitor of Apoptosis Protein 1 (BIRC2)
CML	Chronic myeloid leukemia
CRBN	Cereblon (protein that in humans is encoded by the CRBN gene)
CrkL	Crk-like proto-oncogene, adaptor protein
DFG-in	Asp-Phe-Gly motif in active kinase conformation
DFG-out	Asp-Phe-Gly motif in inactive kinase conformation
DGFR	Discoidin domain receptor growth factor receptor
DMR	Deep molecular response
EMA	European Medicines Agency
FaDu	Human pharyngeal squamous cell carcinoma cell line
FDA	Food and Drug Administration (USA)
FGFR3	Fibroblast growth factor receptor 3
FLT3	Fms-like tyrosine kinase 3
G_0_/G_1_	Gap 0/Gap 1 phases of the cell cycle
GI_50_	50% Growth Inhibition concentration
HAT	Histone acetyltransferase
HBM	Human Body Model
HDAC	Histone deacetylase
IC_50_	Half maximal inhibitory concentration
JAK1-3	Janus kinases 1 to 3
KIT	Stem cell factor receptor (CD117)
LYN	Lyn proto-oncogene, SRC family tyrosine kinase
MCL	Mantle cell lymphoma
MDM2	Mouse double minute 2 homolog
NMPA	National Medical Products Administration (China)
NMR	Nuclear magnetic resonance
NSCLC	Non-small cell lung cancer
OECHEM	OpenEye OEChem Toolkit
PDGFR	Platelet-derived growth factor receptor
PROTAC	Proteolysis-targeting chimera
SAR	Structure–activity relationship
SCC	Squamous cell carcinoma
SRC	Src proto-oncogene, non-receptor tyrosine kinase
STAT5	Signal Transducer and Activator of Transcription 5
TFR	Treatment-free remission
TKI	Tyrosine kinase inhibitor
TRKC	Tropomyosin receptor kinase C
UPS	Ubiquitin–proteasome system
VEGF	Vascular endothelial growth factor
VHL	Von Hippel–Lindau tumor suppressor
Wnt/β-catenin	Wmt/β-catenin signaling pathway
QSAR	Quantitative Structure–Activity Relationship
VHL	von Hippel–Lindau tumor suppressor protein
